# *O*-Alkylated heavy atom carbohydrate probes for protein X-ray crystallography: Studies towards the synthesis of methyl 2-*O*-methyl-L-selenofucopyranoside

**DOI:** 10.3762/bjoc.12.282

**Published:** 2016-12-22

**Authors:** Roman Sommer, Dirk Hauck, Annabelle Varrot, Anne Imberty, Markus Künzler, Alexander Titz

**Affiliations:** 1 Chemical Biology of Carbohydrates, Helmholtz Institute for Pharmaceutical Research Saarland (HIPS), D-66123 Saarbrücken, Germany; 2 Deutsches Zentrum für Infektionsforschung (DZIF), Standort Hannover-Braunschweig, Germany; 3 Centre de Recherche sur les Macromolécules Végétales (CERMAV-UPR5301), CNRS and Université Grenoble Alpes, BP53, F-38041 Grenoble cedex 9, France; 4 Institute of Microbiology, Swiss Federal Institute of Technology (ETH) Zürich, 8093 Zürich, Switzerland

**Keywords:** carbohydrate chemistry, fucose, lectin, selenoglycoside

## Abstract

Selenoglycosides are used as reactive glycosyl donors in the syntheses of oligosaccharides. In addition, such heavy atom analogs of natural glycosides are useful tools for structure determination of their lectin receptors using X-ray crystallography. Some lectins, e.g., members of the tectonin family, only bind to carbohydrate epitopes with O-alkylated ring hydroxy groups. In this context, we report the first synthesis of an *O*-methylated selenoglycoside, specifically methyl 2-*O*-methyl-L-selenofucopyranoside, a ligand of the lectin tectonin-2 from the mushroom *Laccaria bicolor*. The synthetic route required a strategic revision and further optimization due to the intrinsic lability of alkyl selenoglycosides, in particular for the labile fucose. Here, we describe a successful synthetic access to methyl 2-*O*-methyl-L-selenofucopyranoside in 9 linear steps and 26% overall yield starting from allyl L-fucopyranoside.

## Introduction

Since the discovery of seleno mercaptan by Siemens in 1847 [1], organoselenium compounds have attracted high attention. Besides their biological and pharmaceutical relevance, e.g., selenocysteine or ebselen [2–5], selenium-containing derivatives are nowadays used as powerful tools in organic chemistry [6]. Synthetic selenoglycosides are versatile synthons in glycosylation reactions as glycosyl donors for the synthesis of glycosides and oligosaccharides, where their aglycon acts as a leaving group [7–9]. They can be selectively activated due to their enhanced reactivity in the presence of other chalcogen-containing glycosides such as *O*- or *S*-glycosides. By exploiting these properties, one-pot multi-step glycosylation reactions have been developed recently [10–11]. Natural selenosugars, such as methylseleno *N*-acetyl-β-D-glucosamine, have been described in rats as metabolites for detoxifying inorganic selenite intake [12–13].

**Figure 1:**
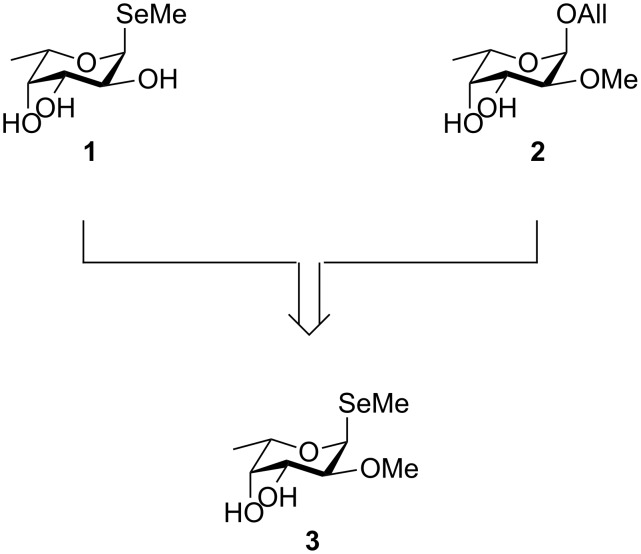
Alkylseleno glycosides, such as **1**, are used as tools for X-ray crystallography of lectins. Some lectins require *O*-alkylation for carbohydrate recognition, e.g., the fungal Lb-Tec2 binds to 2-*O*-methyl fucoside **2**. The heavy-atom probe **3** bearing the required *O*-methylation in position 2 could solve the phase problem for Lb-Tec2 structure determination.

Selenium-containing compounds are also widely used as tools for protein X-ray crystallography in structural biology. The determination of a protein structure depends on the correct phase recovering because only the amplitude of the scattered waves can be directly determined from an X-ray diffraction pattern [14]. Several methods have been developed to solve the so-called “phase problem” such as molecular and isomorphous replacement and multi-wavelength anomalous diffraction (MAD) [14–15]. Molecular replacement is a straight forward method when atomic coordinates of structurally similar proteins are available. For unknown protein structures, heavy atoms, e.g., salts of Hg, Fe, or lanthanides, are incorporated into the protein or the crystal. The changes in intensities of some classes of reflections will enable the localization of heavy metal positions and hence determination of the phases [14–15]. Covalently modified detergents, for example selenium-containing alkyl glycosides, have been used to incorporate heavy atoms into the crystal [16–18]. To overcome problems in locating numerous rather positionally undefined heavy atoms obtained after an unspecific ligand-soaking procedure, new methods were developed. One highly defined method is to directly substitute methionine by selenomethionine in recombinantly expressed proteins [19–20]. On the other hand, the co-crystallization of a protein in complex with selenium-containing ligands can enable the phase determination without the need for recombinant incorporation of amino acid analogues. Buts et al. first reported the use of a selenoglycoside as ligand for a carbohydrate-binding protein, and its use for X-ray crystallographic structure determination [21–22]. This method has now successfully been applied to solve the structure of the bacterial lectins RSL from *Ralstonia solanacearum* [23], BC2L-C from *Burkholderia cenocepacia* [24], and the fungal lectin AFL from *Aspergillus fumigatus* [25] using methyl α-L-selenofucoside (**1**, Figure 1) as heavy atom containing ligand as well as PVL lectin from *Psathyrella velutina* [26] using methyl β-D-selenoGlcNAc. Recently, the crystal structure of human galectin-9 in complex with a selenium-containing lactose disaccharide has been described [27].

In 2014, we have characterized the toxin Tectonin-2 from the mushroom *Laccaria bicolor* (Lb-Tec2) [28], a protein that belongs to the tectonin family of β-propeller lectins and plays an important role in fungal defense against bacteria and nematodes. The determination of the Lb-Tec2 structure by X-ray crystallography promised to be difficult since no suitable model was available for molecular replacement and the protein contains only one methionine and no cysteine residues, which is insufficient to consider incorporation of selenomethionine in the protein for the structure elucidation. Its carbohydrate-binding specificity was determined and a preference for *O-*methylated carbohydrate ligands was demonstrated (e.g., **2**, Figure 1). *O-*Methylation of carbohydrates is a rare modification, but widespread in nature as it has been observed in bacteria, protozoa, animals and plants but not in mammals [29–30]. Besides fungal and animal tectonins that recognize *O*-methylated glycans in pathogens or parasites, numerous other lectins recognize such *O*-alkylated ligands, e.g., the pilus adhesin from *Pseudomonas aeruginosa* PAK [31] or PapG from *Escherichia coli* [32]. In contrast, methylation of lectin ligands can also prevent binding, as observed with *O*-methylated fucose and mannose for *P. aeruginosa* LecB or *B. cenocepacia* BC2L-A [33]. Thus, *O*-methylation of glycans can tune biological recognition events.

In contrast to the literature reports on the synthesis of unmodified seleno glycosides, the synthesis of selectively *O*-alkylated derivatives to study their interactions with lectins has not been described. Here, we report for the first time the synthesis of a selectively ring-substituted methylseleno-fucoside and provide insight into the reactivity and reagent tolerability of seleno-glycosides. The methods described will be useful for application in the synthesis of heavy-atom probes for *O*-alkyl carbohydrate binding lectins.

## Results and Discussion

**Scheme 1:**
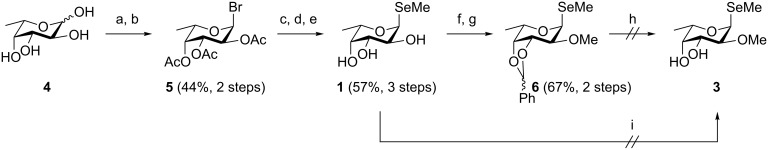
Synthesis of **3** through initial introduction of the seleno aglycon and subsequent *O*-methylation. Reagents and conditions: (a) NaOAc, Ac_2_O, 140 °C, 3 h; (b) TMSBr, CH_2_Cl_2_, 0 °C–rt, 6 h; (c) Me_2_Se_2_, NaBH_4_, MeCN, 90 °C, 1.5 h; (d) BF_3_·OEt_2_, CH_2_Cl_2_, rt, 3 h; (e) NaOMe, MeOH, rt, 1 h; (f) PhCH(OMe)_2_, camphorsulfonic acid, DMF, 50 °C, 20 mbar, 30 min; (g) 1. NaH, DMF, 0 °C, 1 h, 2. MeI, DMF, 0 °C, 10 min; (h) various conditions, see Table 1; (i) *t*-BuOK, MeI, THF, rt, 24 h.

The first synthetic approach towards *O*-methylated selenofucoside **3** was based on the reported syntheses of unmodified methyl α-L-selenofucoside (**1**) [23] and of the reported tectonin ligand allyl 2-*O-*methyl-α-L-fucoside (**2**) [28]. After the introduction of the seleno aglycon, a subsequent selective methylation was expected to lead to the desired derivative **3**. For this purpose, L-fucose (**4**) was fully acetylated and then treated with TMSBr following the protocols from Gilliard et al. [34] to give glycosyl bromide **5** (Scheme 1). The introduction of the methylseleno moiety was performed by nucleophilic substitution of the α-bromide in **5** with methylselenol obtained by in situ reduction of dimethyl diselenide with NaBH_4_ [23]. The obtained crude methyl β-selenofucoside was anomerized under Lewis acid catalysis to give the anomeric mixture in a ratio of α/β = 2:1. After separation of the anomers, pure methyl α-L-selenofucoside (**1**) was finally obtained after deprotection of the α-anomer in 25% over 5 steps from L-fucose (**4**).

A selective methylation of the hydroxy group in position 2 requires prior protection of the *cis*-diol in position 3 and 4 in selenofucoside **1**. The introduction of a 3,4-*O*-benzylidene protecting group using benzaldehyde dimethyl acetal under standard conditions was ineffective and degradation was observed by TLC. However, the formation of the 3,4-*O*-benzylidene acetal could be achieved through in situ evaporation of the reaction byproduct methanol as previously reported by Evans [35] for methyl *O*-glucosides. Using the same conditions, 4,6-benzylidene protection was successfully introduced into a phenylseleno glucoside by Fairbanks et al. [36] without observed degradation at ambient pressure. The increased instability in our system was likely resulting both from the more reactive methyl aglycon and the more reactive fucose carbohydrate part, in analogy to the differences in reactivity of related thioglycosides [37]. Next, the free hydroxy group was methylated using methyl iodide to give derivative **6** in 67% yield over two steps (Scheme 1). To prevent degradation of the labile seleno moiety, the reaction times were kept as short as possible.

Various conditions were tested for the deprotection of **6** to give the desired methyl 2-*O*-methyl-L-selenofucoside (**3**) (Table 1). Under mild acidic conditions (chloroform, Table 1, entry 1) no conversion was observed. Therefore, more acidic conditions were used but treatment of **6** with acetic acid led to degradation (Table 1, entry 2). The isolated reaction products were devoid of the characteristic NMR signals of the methyl aglycon of the selenofucoside at ≈2 ppm (^1^H NMR) and ≈2 ppm (^13^C NMR). To prevent acidic degradation, **6** was treated with palladium on charcoal under a hydrogen atmosphere (Table 1, entries 3–5). In MeOH as the solvent, transglycosylation of the seleno glycoside **6** to its methyl *O*-glycoside was observed (Table 1, entry 3). Changing the solvent to the non-nucleophilic 1,4-dioxane or THF led to either no conversion (dioxane, Table 1, entry 4) or complete degradation and formation of various side products (THF, Table 1, entry 5). Finally, Birch reduction conditions were employed, however, without success leading to degradation of **6** (Table 1, entry 6). Due to the unsuccessful removal of the benzylidene group in **6**, unprotected selenofucoside **1** was directly methylated with MeI (Scheme 1). However, only a slow conversion was observed and various products were detected on TLC lacking predominant formation of only one regioisomer.

**Table 1:** Tested reaction conditions for the deprotection of benzylidene derivative **6**.

Entry	Reaction conditions	Product formation

1	CHCl_3_/H_2_O 30:1, rt, 12 h	–^a^
2	AcOH/H_2_O 4:1, rt, 20 h	degradation^b^
3	10 wt % Pd/C, H_2_, MeOH, rt, 13 h	transglycosylation
4	10 wt % Pd/C, H_2_, 1,4-dioxane, rt, 12 h	–^a^
5	20 wt % Pd/C, H_2_, THF, rt, 12 h	degradation^b^
6	Na, *t*-BuOH, THF, NH_3_, −78 °C, 10 min	degradation^b^

^a^No conversion observed by TLC*. *^b^Degradation was confirmed by NMR spectroscopy of the reaction products where the characteristic peaks for the methyl aglycon of the selenofucoside (≈2 ppm in ^1^H NMR and ≈2 ppm in ^13^C NMR) could not be observed.

**Scheme 2:**
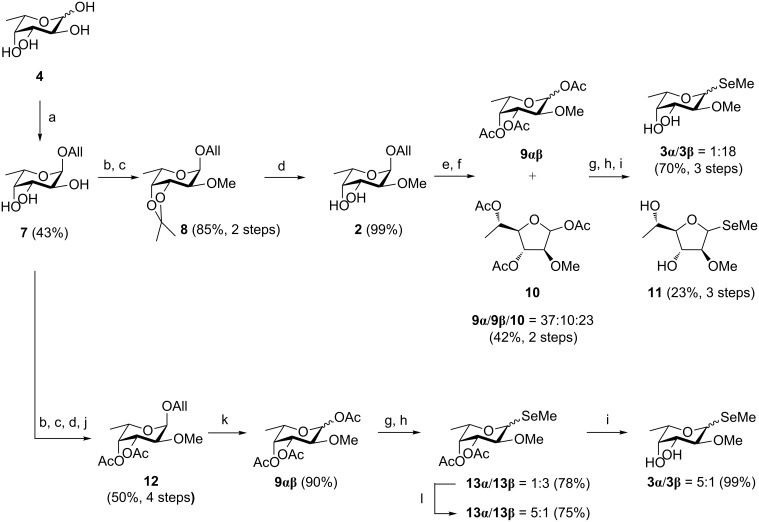
Synthesis of compound **3** via initial selective 2-*O*-methylation followed by the introduction of the seleno aglycon. Reagents and conditions: (a) allyl alcohol, Amberlite IR120 (H^+^), 70 °C, 18 h; (b) 2,2-dimethoxypropane, *p*-toluenesulfonic acid, acetone, rt, 1 h; (c) 1. NaH, DMF, 0 °C, 1 h; 2. MeI, DMF, 0 °C, 30 min; (d) AcOH, H_2_O, 90 °C, 30 min; (e) PdCl_2_, CH_2_Cl_2_, MeOH, rt, 24 h; (f) NaOAc, Ac_2_O, 90 °C, 1.5 h; (g) TMSBr, CH_2_Cl_2_, 0 °C, 2.5 h; (h) (Me_2_Se_2_, NaBH_4_, MeCN, 90 °C, 1 h), CH_2_Cl_2,_ 90 °C 15 min–1 h; (i) NaOMe, MeOH, rt, 30 min–2 h; (j) Ac_2_O, pyridine, 0 °C to rt, 3 h; (k) Ac_2_O, BF_3_·OEt_2_, 0 °C to rt, 17 h; (l) BF_3_·OEt_2_, CH_2_Cl_2_, rt, 20 h.

To overcome the difficulties of the deprotection reaction in the presence of the labile seleno aglycon, the strategy was changed and the seleno moiety was introduced after methylation of fucose in position 2. As in our previous synthesis of 2-*O*-methylated fucoside [28], allyl alcohol was glycosylated with L-fucose (**4**) in a Fischer-type glycosylation and pure allyl α-fucoside (**7**) was obtained after crystallization in 43% yield (Scheme 2). Selective protection of the 3,4-*cis*-diol as an acetonide followed by methylation of the hydroxy group in position 2 yielded derivative **8** in 85% yield over two steps. Then, the acetonide protecting group in **8** was removed by treatment with acetic acid to give **2** [28] in 99% yield. For activation of the anomeric center, the allyl aglycon was subsequently cleaved by palladium-catalyzed transallylation to methanol and the fully acetylated donor **9** was generated in refluxing acetic anhydride and sodium acetate as base.

From this acetylation reaction, a chromatographically inseparable mixture of isomeric per-*O*-acetylated pyranosides **9**α/**9**β and a single furanoside **10** in a ratio of **9**α/**9**β/**10** = 3:1:3 was obtained in 42% yield over the two steps (Scheme 2). The isomeric mixture of acetates was then activated using TMSBr and different retention times of pyranosyl bromides and undesired furanosyl bromides on TLC were observed. However, preparative chromatographic separation failed probably due to an enhanced reactivity of the partially ‘armed’ ether protected halogen glycosides [38] and degradation was observed. Therefore, the glycosylation of methyl selenol using the mixture of donors **9**/**10** was performed first. After Zemplén deprotection of the acetate protecting groups, separation of the unprotected pyranose/furanose isomers was achieved and seleno pyranosides **3** (**3**α/**3**β = 1:18, Scheme 2) as well as one seleno furanoside **11** were obtained over three steps in 70% and 23% yield, respectively.

By this route, the desired seleno glycoside **3** could be successfully synthesized with a high β/α ratio of 18:1. Previously, the tectonins were shown to bind the α-anomer of 2-*O*-methylated fucoside, and natural fucosides are generally α-linked across all kingdoms of life, in glycoproteins, glycolipids, bacterial lipopolysaccharides, or low-molecular weight glycoconjugates such as glycosylated natural products. β-Linked fucosides are rarely observed, for example in the bacterial polysaccharide colanic acid [39–40] or plant natural products [41]. In order to optimize the synthesis of **3** by avoiding the formation of the furanose acetate **10** and increasing the α-selectivity, we revised our synthetic approach. The problematic formation of furanosides has been reported by Kovac et al. for xylose derivatives bearing a benzyl ether protecting group in O-2 position [42]. By varying the reaction conditions during the acetylation step, the authors were able to reduce the formation of furanoses but complete suppression was not achieved. Therefore, our strategy was to block the hydroxy group in position 4 and, thus, to prevent 5-membered ring formation by this group. The *cis*-dihydroxy groups in position 3 and 4 of compound **2** were acetylated (→ **12**) prior to the deprotection of the anomeric center. Following this sequence, allyl 3,4-di-*O-*acetyl-2-*O-*methyl-α-L-fucoside (**12**) was synthesized in 50% yield over four steps from the unprotected allyl fucoside **7** without the need of time-consuming purification of the intermediates. Then, cleavage of the allyl group and subsequent introduction of the anomeric acetate was catalyzed with BF_3_·OEt_2_ in the presence of acetic anhydride. Under these conditions [43] a simultaneous cleavage and in situ acetylation resulted in the pyranose mixture **9**αβ with excellent yield (90%, **9**α/**9**β = 4:1) and prevention of unwanted furanoside side products. The seleno moiety was then introduced as described before and **13**αβ (**13**α**/13**β = 1:3) was obtained in 78% yield. Further Lewis acid catalyzed anomerization improved the ratio towards the α-anomer (**13**α/**13**β = 5:1 after 24 h). This α/β**-**ratio of 5:1 is likely to be the equilibrium of this reaction since it was determined after 2 h and remained constant (see Supporting Information File 1). Finally, the desired methyl 2-*O*-methyl selenofucoside (**3**) could be isolated after Zemplén deprotection in 99% yield and a ratio of α/β = 5:1.

## Conclusion

Unsubstituted seleno glycosides are used for structure determination of complexes with protein receptors. The synthesis of *O*-methylated analogs has been not reported to date, despite their importance for many lectins. The first synthetic route starting from the known methyl selenofucoside **1** failed due to the rather high stability of the 3,4-benzylidene protecting group required for the selective *O*-methylation in position 2. Because the instability of the selenium aglycon was the limiting factor, we then first selectively methylated the protected fucose in position 2 and introduced the selenium moiety subsequently. Despite the fact that methyl 2-*O*-methyl-L-selenofucoside was obtained following this synthetic route, the yield was reduced due to extensive furanoside formation as a result of the methylation in position 2. Optimization of the protecting group strategy finally yielded the desired methyl 2-*O*-methyl L-selenofucoside (**3**) in 26% yield over nine linear steps from allyl L-fucoside. We have soaked compound **3** into crystals of tectonin which allowed phasing by MAD and solution of the structure at 1.65 Angstroms and these details will be reported in due course.

## Supporting Information

File 1 Chemical synthesis, ^1^H NMR and ^13^C NMR traces of synthesized compounds.
