# Supplementary material for: O-Alkylated heavy atom carbohydrate probes for protein X-ray crystallography: Studies towards the synthesis of methyl 2-O-methyl-L-selenofucopyranoside
Source: Beilstein J Org Chem. 2016 Dec 22;12:2828–33. doi: 10.3762/bjoc.12.282 (PMC5238581; doi:10.3762/bjoc.12.282)
Supplement: File 1 — Chemical synthesis, 1H NMR and 13C NMR traces of synthesized compounds. [file Beilstein_J_Org_Chem-12-2828-s001.pdf]

Supporting Information

for

O-Alkylated heavy atom carbohydrate probes for protein X-ray crystallography: Studies towards the synthesis of methyl 2-*O*-methyl-L-selenofucopyranoside

Roman Sommer[1,2], Dirk Hauck[1,2], Annabelle Varrot[3], Anne Imberty[3], Markus Künzler[4], Alexander Titz[1,2*]

Address: [1]Chemical Biology of Carbohydrates, Helmholtz Institute for Pharmaceutical Research Saarland (HIPS), D-66123 Saarbrücken, Germany, [2]Deutsches Zentrum für Infektionsforschung (DZIF), Standort Hannover-Braunschweig, Germany, [3]Centre de Recherche sur les Macromolécules Végétales (CERMAV-UPR5301), CNRS and Université Grenoble Alpes, BP53, F-38041 Grenoble cedex 9, France and [4]Institute of Microbiology, Swiss Federal Institute of Technology (ETH) Zürich, 8093 Zürich, Switzerland

Email: Alexander Titz - alexander.titz@helmholtz-hzi.de

*Corresponding author

Chemical synthesis, ^{1}H NMR and ^{13}C NMR traces of synthesized compounds

Experimental

Chemical synthesis

Silica gel 60-coated aluminum sheets containing fluorescence indicator (Merck KGaA, Darmstadt, Germany) were used for thin layer chromatography (TLC). UV light (254 nm) and aqueous $KMnO_4$ solution or a molybdate solution (a 0.02 M solution of ammonium cerium sulfate dihydrate and ammonium molybdate tetrahydrate in aqueous 10% H_2SO_4) were used for development. Preparative medium pressure liquid chromatography (MPLC) was performed on a Teledyne Isco Combiflash Rf200 system using pre-packed silica gel 60 columns from Interchim. Optical rotation was measured using a P-2000 polarimeter (Jasco, Gross-Umstadt, Germany) at 589 nm. Nuclear magnetic resonance (NMR) spectroscopy was performed on a Bruker Avance III 500 UltraShield spectrometer at 500 MHz (^{1}H) or 126 MHz (^{13}C). Chemical shifts are given in ppm and were calibrated on residual solvent peaks as internal standard [1]. Multiplicities were specified as s (singlet), d (doublet), t (triplet) or m (multiplet). The signals were assigned with the help of ^{1}H,^{1}H-COSY, DEPT-135-edited ^{1}H,^{13}C-HSQC and ^{1}H,^{13}C-HMBC experiments. Mass spectra were obtained on a Bruker amaZon SL for low resolution or on a Bruker maxis 4G hr-QqToF spectrometer for high resolution, and the data were analyzed using DataAnalysis (Bruker Daltonics, Bremen, Germany). Commercial chemicals and solvents were used without further purification. Deuterated solvents were purchased from Eurisotop (Saarbrücken, Germany).

Methyl 3,4-*O*-benzylidene-2-*O*-methyl-α-L-selenofucopyranoside (6). Methyl α-L-selenofucoside [2] (**1**, 40 mg, 0.16 mmol) was stirred in dry DMF (2 mL) in the presence of benzaldehyde dimethyl acetal (250 µL, 1.64 mmol) and camphorsulfonic acid (3.7 mg, 0.02 mmol) at 50 °C in vacuo (20 mbar) for 30 min. Then, the reaction mixture was cooled to 0 °C, dry DMF (1 mL) and NaH (77 mg, 1.92 mmol, 60 wt % in mineral oil) were added and

the reaction mixture was stirred for 1 h at 0 °C. MeI (120 µL, 1.92 mmol) was added and the reaction was protected from light and stirred for 10 min. The colorless reaction mixture was then quenched at 0 °C with aqueous saturated NH_4Cl solution and extracted with EtOAc (3 × 15 mL). The combined organic layers were dried over Na_2SO_4, filtered and the volatiles were removed in vacuo. After MPLC purification (petrol ether to petrol ether/EtOAc 7:1), the title compound **6** was obtained as a colorless oil (37 mg, 0.11 mmol, 67%, 2 steps) as a mixture of the benzylidene diastereomers in a ratio of *R*/*S* = 10:6. The assignment of the stereochemistry of the benzylidene diastereomers was deduced from the 1-deoxy-fucose analog previously reported by us [3]. *S*-isomer: ^{1}H NMR (500 MHz, CH_2Cl_2-d_2) δ 7.55 – 7.50 (m, 1H, ArC<u>H</u>), 7.47 – 7.35 (m, 4H, ArC<u>H</u>), 5.87 (s, 1H, OOC<u>H</u>Ph), 5.73 (d, J = 5.3 Hz, 1H, H-1), 4.34 – 4.30 (m, 2H, H-3, H-5), 4.15 (dd, J = 6.6, 2.4 Hz, 1H, H-4), 3.63 (dd, J = 5.9, 5.3 Hz, 1H, H-2), 3.44 (s, 3H, OC<u>H</u>$_3$), 1.97 (s, 3H, SeC<u>H</u>$_3$), 1.35 (d, J = 6.7 Hz, 3H, H-6). ^{13}C NMR (126 MHz, CH_2Cl_2-d_2) δ 138.2 (ArC), 129.9 (ArCH), 128.9 (ArCH), 127.4 (ArCH), 104.4 (OO<u>C</u>HPh), 80.8 (C-1), 79.2 (C-2), 78.1 (C-4), 75.3 (C-3), 66.1 (C-5), 58.8 (O<u>C</u>H$_3$), 16.5 (<u>C</u>H$_3$), 2.2 (Se<u>C</u>H$_3$). *R*-isomer: ^{1}H NMR (500 MHz, CH_2Cl_2-d_2) δ 7.55 – 7.50 (m, 1H, ArC<u>H</u>), 7.47 – 7.35 (m, 4H, ArC<u>H</u>), 6.15 (s, 1H, OOC<u>H</u>Ph), 5.79 (d, J = 5.4 Hz, 1H, H-1), 4.42 (dd, J = 6.4, 5.9 Hz, 1H, H-3), 4.29 – 4.25 (m, 1H, H-5), 4.12 (dd, J = 5.8, 2.2 Hz, 1H, H-4), 3.72 (dd, J = 6.5, 5.4 Hz, 1H, H-2), 3.50 (s, 3H, OC<u>H</u>$_3$), 1.96 (s, 3H, SeC<u>H</u>$_3$), 1.34 (d, J = 6.7 Hz, 3H, H-6). ^{13}C NMR (126 MHz, CH_2Cl_2-d_2) δ 139.8 (ArC), 129.6 (ArCH), 128.9 (ArCH), 126.8 (ArCH), 103.9 (OO<u>C</u>HPh), 80.7 (C-1), 77.4 (C-2), 76.8 (C-4), 76.2 (C-3), 66.4 (C-5), 58.8 (O<u>C</u>H$_3$), 16.6 (<u>C</u>H$_3$), 2.1 (Se<u>C</u>H$_3$). ESI-MS m/z: $[M + MeCN + Na]^+$ Calcd for $C_{17}H_{23}NO_4SeNa$ 408.1; Found 408.1.

Allyl 3,4-*O*-isopropylidene 2-*O*-methyl-α-L-fucopyranoside (8). Allyl α-L-fucopyranoside [4] (**7**, 0.60 g, 2.94 mmol) was stirred in acetone (6 mL) in the presence of *p*-toluenesulfonic acid (cat.) and 2,2-dimethoxypropane (370 µL, 2.90 mmol) at rt for 1 h. The mixture was

neutralized with Amberlite IRA-400(OH), filtered and the volatiles were removed in vacuo. The light brown solid was dissolved in dry DMF (9 mL) and cooled to 0 °C. NaH (36 mg, 2.9 mmol, 60 wt % in mineral oil) was then added and the reaction mixture was stirred for 1 h at 0 °C. Methyl iodide (183 µL, 9.0 mmol) was added drop wise and stirring was continued for 30 min. The reaction mixture was quenched with aqueous satd. NH_4Cl solution (2 mL) and extracted with EtOAc (3 × 15 mL). The combined organic layers were dried over Na_2SO_4, filtered and the volatiles were removed in vacuo. MPLC purification (petrol ether to petrol ether/EtOAc 4:1, with 1% Et_3N) yielded the title compound **8** as an oil (0.65 g, 2.50 mmol, 85%, 2 steps). ^{1}H NMR (500 MHz, MeOH-*d₄*) δ 5.93 (dddd, J = 17.3, 10.5, 6.0, 5.2 Hz, 1H, $OCH_2C\underline{H}CH_2$), 5.32 (ddt, J = 17.3, 1.7, 1.7 Hz, 1H, $OCH_2CHC\underline{H_2}$), 5.18 (ddt, J = 10.4, 1.9, 1.3 Hz, 1H, $OCH_2CHC\underline{H_2}$), 4.92 (d, J = 3.6 Hz, 1H, H-1), 4.20 – 4.08 (m, 4H, H-3, H-4, H-5, $OC\underline{H_2}CHCH_2$), 4.00 (ddt, J = 13.1, 6.0, 1.4 Hz, 1H, $OC\underline{H_2}CHCH_2$), 3.48 (s, 3H, $OC\underline{H_3}$), 3.34 (dd, J = 7.9, 3.5 Hz, 1H, H-2), 1.50 (s, 3H, $C(C\underline{H}_3)_2$), 1.33 (br d, J = 0.7 Hz, 3H, $C(C\underline{H}_3)_2$), 1.29 (d, J = 6.5 Hz, 3H, H-6); ^{13}C NMR (126 MHz, MeOH-*d₄*) δ 135.3 ($OCH_2\underline{C}HCH_2$), 117.7 ($OCH_2CH\underline{C}H_2$), 109.9 ($\underline{C}(CH_3)_2$), 96.8 (C-1), 80.7 (C-2), 77.4, 77.0, 69.4 ($O\underline{C}H_2CHCH_2$), 64.6 (C-5), 58.8 ($O\underline{C}H_3$), 28.6 ($C(\underline{C}H_3)_2$), 26.6($C(\underline{C}H_3)_2$), 16.5(C-6). ESI-MS calcd. $C_{13}H_{22}NaO_5^+$: 281.1; found: 280.8. HRMS (ESI-TOF) m/z: $[M + Na]^+$ Calcd for $C_{13}H_{22}O_5Na$ 281.1359; Found 281.1363. $[\alpha]_D^{23}$ -152 (c 0.2, MeCN).

1,3,4-Tri-*O*-acetyl-2-*O*-methyl-L-fucopyranose (9αβ) and 1,3,5-tri-*O*-acetyl-2-*O*-methyl-L-fucofuranose (10). Acetonide **8** (3.35 g, 13.0 mmol) was dissolved in HOAc and stirred at 90 °C. Several portions H_2O were added during 30 min until the conversion was completed. The volatiles were removed in vacuo and after co-evaporation with toluene (3 × 15 mL), the crude product was purified by MPLC (CH_2Cl_2 to CH_2Cl_2/MeOH 20:1) and partially unprotected **2** (2.83 g, 13.0 mmol, 99%) was obtained. Subsequently, **2** (2.83 g, 13.0 mmol) was dissolved in a mixture of MeOH (20 mL) and CH_2Cl_2 (20 mL). $PdCl_2$ (576 mg, 3.25

mmol) was added and the reaction mixture was stirred under a nitrogen atmosphere at rt for 24 h. The orange suspension was filtered over celite and the volatiles were removed in vacuo. MPLC purification (CH_2Cl_2 to CH_2Cl_2/MeOH 10:1) yielded 2-*O*-methyl-L-fucose (1.0 g, 5.6 mmol, 43%) which was directly acetylated in Ac_2O (30 mL) and NaOAc (500 mg, 6.17 mmol) at 90 °C for 1.5 h. Then, the reaction was cooled to rt and neutralized with Amberlite IR120 (H^+), filtered over celite and the volatiles were removed in vacuo. After purification by MPLC (petrol ether to petrol ether/EtOAc 3:1) the inseparable isomeric mixture of peracetylated pyranoses **9**α/**9**β containing one single furanose **10** were obtained (716 mg, 2.35 mmol, 42%) in a ratio of **9**α/**9**β/**10** = 37:10:23 as an oil. ^{1}H NMR and ^{13}C NMR assignment of **9**α and **9**β see below for the synthesis of **9** from **12**. Selected NMR data for 2-*O*-methyl-1,3,5-tri-*O*-acetyl-L-fucofuranose (**10**): ^{1}H NMR (500 MHz, $CHCl_3$-d_1) δ 6.20 (s, 1H, H-1), 5.14 (qd, *J* = 4.7, 6.5 Hz, 1H, H-5), 5.03 – 5.01 (m 1H), 3.79 – 3.78 (m 1H), 1.28 (d, *J* = 1.28, 3H, H-6). The assignment of the anomeric configuration of furanose triacetate **10** was not attempted due to the absence of the second anomer in the furanose series. In case both furanoses are present, the configuration could be assigned by NMR as published for related tetraacetates [5].

Methyl 2-*O*-methyl-L-1-seleno-fucopyranoside (3αβ) and methyl 2-*O*-methyl-L-1-seleno-fucofuranoside (11). The isomeric peracetylated mixture of pyranoses and furanoses **9**α/**9**β/**10** (100 mg, 0.32 mmol) was dissolved in dry CH_2Cl_2 (2 mL) and cooled to 0 °C. Trimethylsilyl bromide (100 μL, 0.76 mmol) was added drop wise and the reaction mixture was stirred at 0 °C for 2.5 h. This glycosyl bromide solution was transferred to a solution of freshly prepared methylselenol (320 μL Me_2Se_2, 213 mg $NaBH_4$, 10 mL MeCN, see Kostlanova et al.) and stirred at 90 °C for further 15 min. The suspension was poured into aqueous HCl (20 mL, 1 M) at 0 °C, the phases were separated and the aqueous phase was extracted with CH_2Cl_2 (3 × 20 mL). The combined organic layers were dried over Na_2SO_4

and the volatiles were removed in vacuo. After purification by MPLC (petrol ether to petrol ether/EtOAc 3:1) the acetylated selenofucoside (75 mg, 0.22 mmol) was obtained and directly deacetylated with NaOMe (140 µL, 30% in MeOH) in MeOH (6 mL) at rt for 30 min. The reaction mixture was neutralized with Amberlite IR120 (H^+), filtered over celite and the volatiles were removed in vacuo. Purification by MPLC (petrol ether to petrol ether/THF 2:1) yielded two separate fractions: First, methyl 2-*O*-methyl-L-1-seleno-fucofuranoside (**11**, one single unassigned anomer; 11.8 mg, 0.05 mmol) was obtained as colorless amorphous solid and then methyl 2-*O*-methyl-L-1-seleno-fucopyranoside (**3**αβ, ratio **3**α/**3**β = 1:18, 35.7 mg, 0.14 mmol) as colorless amorphous solid. Analytical data for methyl 2-*O*-methyl-β-L-1-seleno-fucopyranoside (**3**β) are described below. Analytical data for methyl 2-*O*-methyl-L-1-seleno-fucofuranoside (**11**): ^{1}H NMR (500 MHz, MeOH-d_4) δ 5.75 (d, J = 5.4 Hz, 1H, H-1), 4.08 (t, J = 6.0 Hz, 1H, H-3), 3.93 (dq, J = 7.4, 6.5 Hz, 1H, H-5), 3.86 (t, J = 5.6 Hz, 1H, H-2), 3.55 (dd, J = 7.3, 6.3 Hz, 1H, H-4), 3.43 (s, 3H, $OC\underline{H}_3$), 2.05 (s, 3H, $SeC\underline{H}_3$), 1.17 (d, J = 6.5 Hz, 3H, H-6). ^{13}C NMR (126 MHz, MeOH-d_4) δ 89.7 (C-2/C-4), 89.7 (C-2/C-4), 84.1 (C-1), 76.9 (C-3), 69.9 (C-5), 58.9 ($O\underline{C}H_3$), 19.1 (C-6), 2.3 ($Se\underline{C}H_3$). ESI-MS calcd. $C_8H_{16}NaO_4Se^+$: 279.0; found: 278.7. HRMS (ESI-TOF) m/z: $[M + Na]^+$ calcd for $C_8H_{16}O_4SeNa$ 279.0106; found 279.0106. $[\alpha]_D^{23}$ -115 (c 0.2, MeCN).

Allyl 3,4-di-*O*-acetyl-2-*O*-methyl-α-L-fucopyranoside (12). Allyl 2-*O*-methyl-α-L-fucopyranoside (**2**) was synthesized from allyl fucopyranoside (**7**) as described before for its synthesis from **8**, however, without purification of the intermediates. Subsequently, crude derivative **2** (2.95 g, 13.5 mmol) was dissolved in pyridine (100 mL) and Ac_2O (35.7 mL, 378 mmol) was added drop wise at 0 °C. The reaction mixture was allowed to warm to rt and stirred for 3 h. Then, it was poured on ice and extracted with EtOAc (3 × 100 mL). The combined organic layers were dried over Na_2SO_4, filtered and the volatiles were removed in vacuo. After purification by MPLC (petrol ether/EtOAc, gradient 10–90%) pure **12** (2.0 g,

6.61 mmol, 50% over 4 steps) was obtained as colorless amorphous solid. ^{1}H NMR (500 MHz, MeOH-d_4) δ 5.97 (dddd, *J* = 17.2, 10.4, 6.1, 5.2 Hz, 1H, OCH_2CHCH_2), 5.35 (dq, *J* = 17.2, 1.7 Hz, 1H, OCH_2CHCH_2), 5.25 – 5.14 (m, 3H, OCH_2CHCH_2, H-3, H-4), 5.09 (d, *J* = 3.6 Hz, 1H, H-1), 4.21 (dddd, *J* = 13.1, 5.2, 1.3 Hz, 1H, OCH_2CHCH_2), 4.18 – 4.12 (m, 1H, H-5), 4.07 (ddt, *J* = 13.1, 6.1, 1.4 Hz, 1H, OCH_2-allyl), 3.66 (dd, *J* = 10.4, 3.7 Hz, 1H, H-2), 3.43 (s, 3H, OCH_3), 2.14 (s, 3H, Ac), 1.98 (s, 3H, Ac), 1.10 (d, *J* = 6.6 Hz, 3H, H-6). ^{13}C NMR (126 MHz, MeOH-d_4) δ 172.3 (CO), 171.9 (CO), 135.3 (OCH_2CHCH_2), 117.9 (OCH_2CHCH_2), 96.9 (C-1), 76.7 (C-2), 72.8 (C-3/C-4), 71.5 (C-3/C-4), 69.6 (OCH_2CHCH_2), 65.7 (C-5), 58.6 (OCH_3), 20.8 ($COCH_3$), 20.5 ($COCH_3$), 16.1 (C-6). ESI-MS calcd. $C_{14}H_{22}NaO_7^{+}$: 325.1; found: 324.8. HRMS (ESI-TOF) m/z: $[M + Na]^+$ calcd for $C_{14}H_{22}O_7Na$ 325.1258; found 325.1261. $[\alpha]_D^{23}$ -191 (c 0.2, MeCN).

Synthesis of 1,3,4-tri-*O*-acetyl 2-*O*-methyl-L-fucopyranose (9) from 12. Allyl glycoside **12** (1.00 g, 3.31 mmol) was dissolved in Ac_2O (10 mL) and cooled to 0 °C. Then, $BF_3{\cdot}OEt_2$ (180 µL, 1.46 mmol) in Ac_2O (1 mL) was added drop wise under stirring at 0 °C. The reaction mixture was allowed to warm to rt and after 17 h the reaction was stopped by pouring the mixture on ice. After extraction with EtOAc (3 × 50 mL), the combined organic layers were washed with aqueous satd. $NaHCO_3$ solution, dried over Na_2SO_4, filtered and the volatiles were removed in vacuo. After purification by MPLC (petrol ether/EtOAc 7:3), the title compound was obtained as an anomeric mixture in a ratio of **9**α/**9**β = 3.6:1 (909 mg, 90%). NMR-data for **9**α: ^{1}H NMR (500 MHz, $CHCl_3$-d_1) δ 6.41 (d, *J* = 3.7 Hz, 1H, H-1), 5.29 (dd, *J* = 3.4, 1.4 Hz, 1H, H-4), 5.21 (dd, *J* = 10.5, 3.3 Hz, 1H, H-3), 4.20 (dq, *J* = 6.8, 1.4 Hz, 1H, H-5), 3.70 (dd, *J* = 10.6, 3.7 Hz, 1H, H-2), 3.41 (s, 3H, OCH_3), 2.17 (s, 3H, Ac), 2.14 (s, 3H, Ac), 2.02 (s, 3H, Ac), 1.12 (d, *J* = 6.5 Hz, 3H, H-6). ^{13}C NMR (126 MHz, $CHCl_3$-*d*) δ 170.6 (CO), 170.3 (CO), 169.6 (CO), 89.8 (C-1), 74.6, 71.0, 69.9, 67.2, 59.1 (OCH_3), 21.2 ($COCH_3$), 21.0 ($COCH_3$), 20.8 ($COCH_3$), 16.1 (C-6). NMR-data for **9**β: ^{1}H NMR (500 MHz,

$CHCl_3$-*d₁*) δ 5.55 (d, *J* = 8.2 Hz, 1H, H-1), 5.22 (dd, *J* = 3.5, 1.1 Hz, 1H, H-4), 4.94 (dd, *J* = 10.1, 3.5 Hz, 1H, H-3), 3.89 (qd, *J* = 6.4, 1.1 Hz, 1H, H-5), 3.49 (dd, *J* = 10.2, 8.2 Hz, 1H, H-2), 3.48 (s, 3H, $OC\underline{H}_3$), 2.16 (s, 3H, Ac), 2.03 (s, 3H, Ac), 1.18 (d, *J* = 6.4 Hz, 3H, H-6). ^{13}C NMR (126 MHz, $CHCl_3$-*d*) δ 170.6 (CO), 170.1 (CO), 169.2 (CO), 94.1 (C-1), 77.0, 73.1, 70.5, 70.0, 60.9 ($O\underline{C}H_3$), 21.2 ($CO\underline{C}H_3$), 20.9 ($CO\underline{C}H_3$), 20.8 ($CO\underline{C}H_3$), 16.1 (C-6). ESI-MS of anomeric mixture **9**αβ: calcd. $C_{13}H_{20}NaO_8^+$: 327.1; found: 326.8. HRMS (ESI-TOF) m/z: $[M + Na]^+$ calcd for $C_{13}H_{20}O_8Na$ 327.1050; found 327.1052.

Methyl 3,4-di-*O*-acetyl-2-*O*-methyl-L-1-seleno-fucopyranoside (13). 1,3,4-tri-*O*-acetyl-2-*O*-methyl-L-fucopyranose (**9**αβ, 200 mg, 0.65 mmol) was dissolved in dry CH_2Cl_2 (3 mL) under a nitrogen atmosphere and cooled to 0 °C. Trimethylsilyl bromide (212 µL, 1.97 mmol) was added drop wise, and the reaction was allowed to warm to rt under stirring which was continued for 16 h. In a separate flask, Me_2Se_2 (370 µL, 1.97 mmol) in dry MeCN (20 mL) was treated with NaH (223 mg, 5.90 mmol, 60 wt % in mineral oil) at 90 °C for 1 h. One more portion of Me_2Se_2 (370 µL, 1.97 mmol) was added. Then, the glycosyl bromide solution was transferred to the methylselenol and after stirring for 1 h at 90 °C, the suspension was poured into cooled (4 °C) aqueous HCl (1 M, 30 mL). The mixture was extracted with CH_2Cl_2 (3 × 30 mL), the combined organic layers were dried over Na_2SO_4 filtered and the volatiles were removed in vacuo. After purification by MPLC (petrol ether to petrol ether/EtOAc 3:1), the title compound **13**αβ (ratio α/β = 1:1.6) was obtained as a colorless oil (173 mg, 0.51 mmol, 78%, 2 steps). NMR-data for **13**α: ^1H NMR (500 MHz, MeOH-*d₄*) δ 5.99 (d, *J* = 5.5 Hz, 1H, H-1), 5.24 – 5.21 (m, 1H, H-4), 5.03 (dd, *J* = 10.2, 3.5 Hz, 1H, H-3), 4.41 – 4.34 (m, 1H, H-5), 3.80 (dd, *J* = 10.2, 5.4 Hz, 1H, H-2), 3.40 (s, 3H, $OC\underline{H}_3$), 2.14 (s, 3H, Ac), 1.97 (s, 3H, Ac), 1.90 (s, 3H, $SeC\underline{H}_3$), 1.14 (d, *J* = 6.5 Hz, 3H, H-6). ^{13}C NMR (126 MHz, MeOH-*d₄*) δ 172.3 (CO), 171.9 (CO), 81.5 (C-1), 76.5 (C-2), 72.7 (C-3), 72.1 (C-4), 67.4 (C-5), 57.9 ($O\underline{C}H_3$), 20.7 ($CO\underline{C}H_3$), 20.5 ($CO\underline{C}H_3$), 16.3 (C-6), 1.1 ($Se\underline{C}H_3$). NMR-data

for **13β**: ^{1}H NMR (500 MHz, MeOH-*d*$_4$) δ 5.24 – 5.21 (m, 1H, H-4), 4.94 (dd, *J* = 9.7, 3.4 Hz, 1H, H-3), 4.64 (d, *J* = 9.8 Hz, 1H, H-1), 3.87 (qd, *J* = 6.4, 1.1 Hz, 1H, H-5), 3.51 (s, 3H, OC<u>H</u>$_3$), 3.43 (t, *J* = 9.7 Hz, 1H, H-2), 2.15 (s, 3H, Ac), 2.12 (s, 3H, SeC<u>H</u>$_3$), 2.01 (s, 3H, Ac), 1.13 (s, 1H, d, *J* = 6.5 Hz, 3H, H-6). ^{13}C NMR (126 MHz, MeOH-*d*$_4$) δ 172.3 (CO), 171.7 (CO), 79.4 (C-2), 79.3 (C-1), 75.8 (C-3), 74.9 (C-5), 72.5 (C-4), 61.0 (O<u>C</u>H$_3$), 20.8 (CO<u>C</u>H$_3$), 20.5 (CO<u>C</u>H$_3$), 16.6 (C-6), 2.3 (Se<u>C</u>H$_3$). ESI-MS for **13αβ** calcd. $C_{12}H_{20}NaO_6Se^+$: 363.0; found: 362.6. HRMS (ESI-TOF) m/z: $[M + Na]^+$ calcd for $C_{12}H_{20}O_6SeNa$ 363.0317; found 363.0319.

Methyl 2-*O*-methyl-L-1-seleno-fucopyranoside (3αβ). Acetylated **13αβ** was isomerized under Lewis acid catalysis to the α-anomer following the procedure described by Kostlanova et al. and a ratio of **13α**/**13β** = 5:1 was obtained after 24 h reaction time. This mixture of **13αβ** (38 mg, 0.112 mmol) was dissolved in dry MeOH (4 mL), NaOMe (20 µL, 30% in MeOH) was added and the solution was stirred at rt for 2 h. The reaction mixture was neutralized with Amberlite IR120 (H^+), filtered over celite and the volatiles were removed in vacuo. Purification by MPLC (CH_2Cl_2 to CH_2Cl_2/MeOH 15:1) yielded **3αβ** (28.4 mg, 0.111 mmol, 99%) as colorless oil in an anomeric ratio of 5:1 (α/β). Analytical data for **3α**: ^{1}H NMR (500 MHz, MeOH-*d*$_4$) δ 5.88 (d, *J* = 5.0 Hz, 1H, H-1), 4.15 (br q, *J* = 6.8 Hz, 1H, H-5), 3.68 – 3.66 (m, 1H, H-4), 3.65 – 3.60 (m, 2H, H-3, H-2), 3.43 (s, 3H, OC<u>H</u>$_3$), 1.86 (s, 3H, SeC<u>H</u>$_3$), 1.24 (d, *J* = 6.6 Hz, 3H, H-6). ^{13}C NMR (126 MHz, MeOH-*d*$_4$) δ 81.9 (C-1), 79.3 (C-2), 73.0 (C-4), 72.3 (C-3), 69.1 (C-5), 57.8 (O<u>C</u>H$_3$), 16.7 (C-6), 1.0 (Se<u>C</u>H$_3$). Analytical data for **3β**: ^{1}H NMR (500 MHz, MeOH-*d*$_4$) δ 4.45 (d, *J* = 9.8 Hz, 1H, H-1), 3.63 (dd, *J* = 3.5, 1.1 Hz, 1H, H-4), 3.58 (qd, *J* = 6.5, 1.1 Hz, 1H, H-5), 3.57 (s, 3H, OC<u>H</u>$_3$), 3.50 (dd, *J* = 9.2, 3.4 Hz, 1H, H-3), 3.27 (t, *J* = 9.5 Hz, 1H, H-2), 2.08 (s, 3H, SeC<u>H</u>$_3$), 1.23 (d, *J* = 6.5 Hz, 3H, H-6). ^{13}C NMR (126 MHz, MeOH-*d*$_4$) δ 82.2 (C-2), 80.0 (C-1), 77.1 (C-5), 76.2 (C-3), 73.5 (C-4), 61.0

(O$\underline{C}H_3$), 16.9 (C-6), 2.3 (Se$\underline{C}H_3$). ESI-MS for **3**αβ calcd. $C_8H_{16}NaO_4Se^+$: 279.0; found: 278.7.

HRMS (ESI-TOF) m/z: $[M + Na]^+$ calcd for $C_8H_{16}O_4SeNa$ 279.0106; found 279.0109.

References

1. Gottlieb, H. E.; Kotlyar, V.; Nudelman, A. *J. Org. Chem.* **1997**, *62*, 7512-7515.
2. Kostlánová, N.; Mitchell, E. P.; Lortat-Jacob, H.; Oscarson, S.; Lahmann, M.; Gilboa-Garber, N.; Chambat, G.; Wimmerová, M.; Imberty, A. *J. Biol. Chem.* **2005**, *280*, 27839-27849.
3. Beshr, G.; Sommer, R.; Hauck, D.; Siebert, D. C. B.; Hofmann, A.; Imberty, A.; Titz, A. *Med. Chem. Commun.* **2016**, *7*, 519-530.
4. Wohlschlager, T.; Butschi, A.; Grassi, P.; Sutov, G.; Gauss, R.; Hauck, D.; Schmieder, S. S.; Knobel, M.; Titz, A.; Dell, A.; Haslam, S. M.; Hengartner, M. O.; Aebi, M.; Künzler, M. *Proc. Natl. Acad. Sci. U. S. A.* **2014**, *111*, E2787-96.
5. Prihar, H.; Tsai, J.; Wanamaker, S.; Duber, S.; Behrman, E. *Carbohydr. Res.* **1977**, *56*, 315-32

^{1}H NMR and ^{13}C NMR traces of synthesized compounds

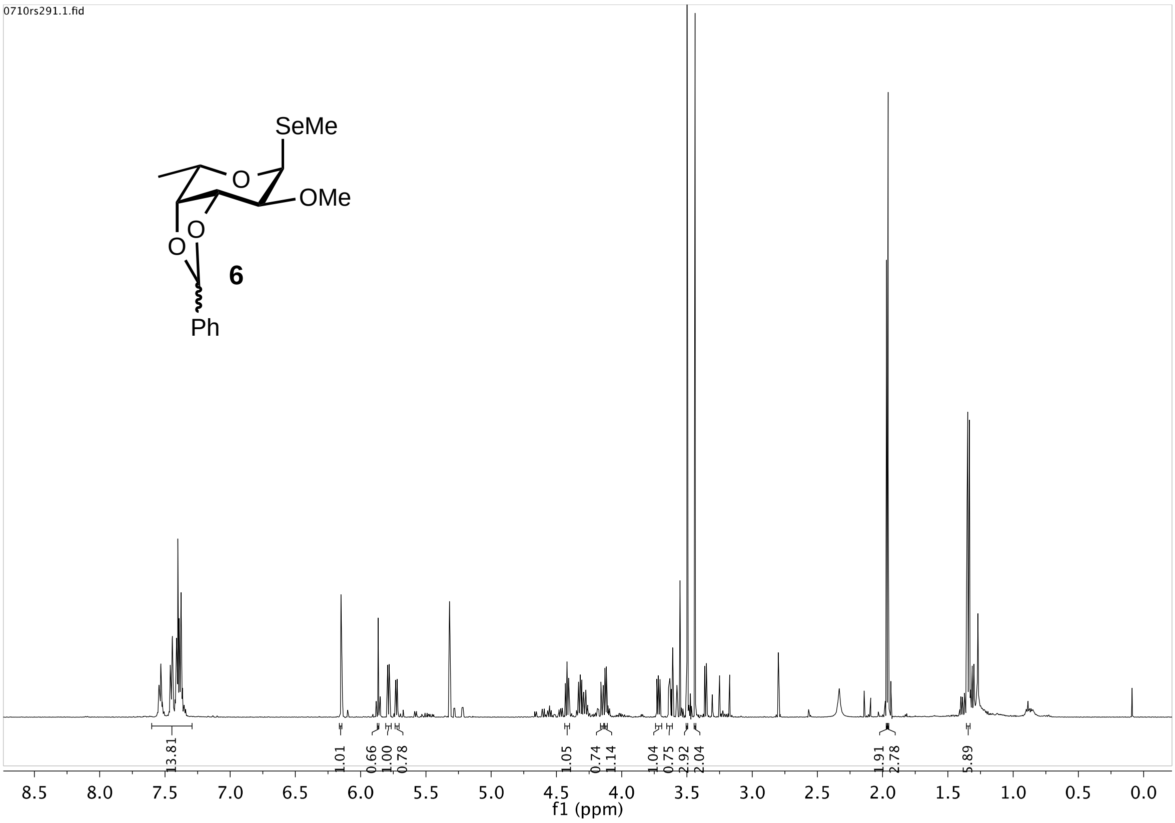

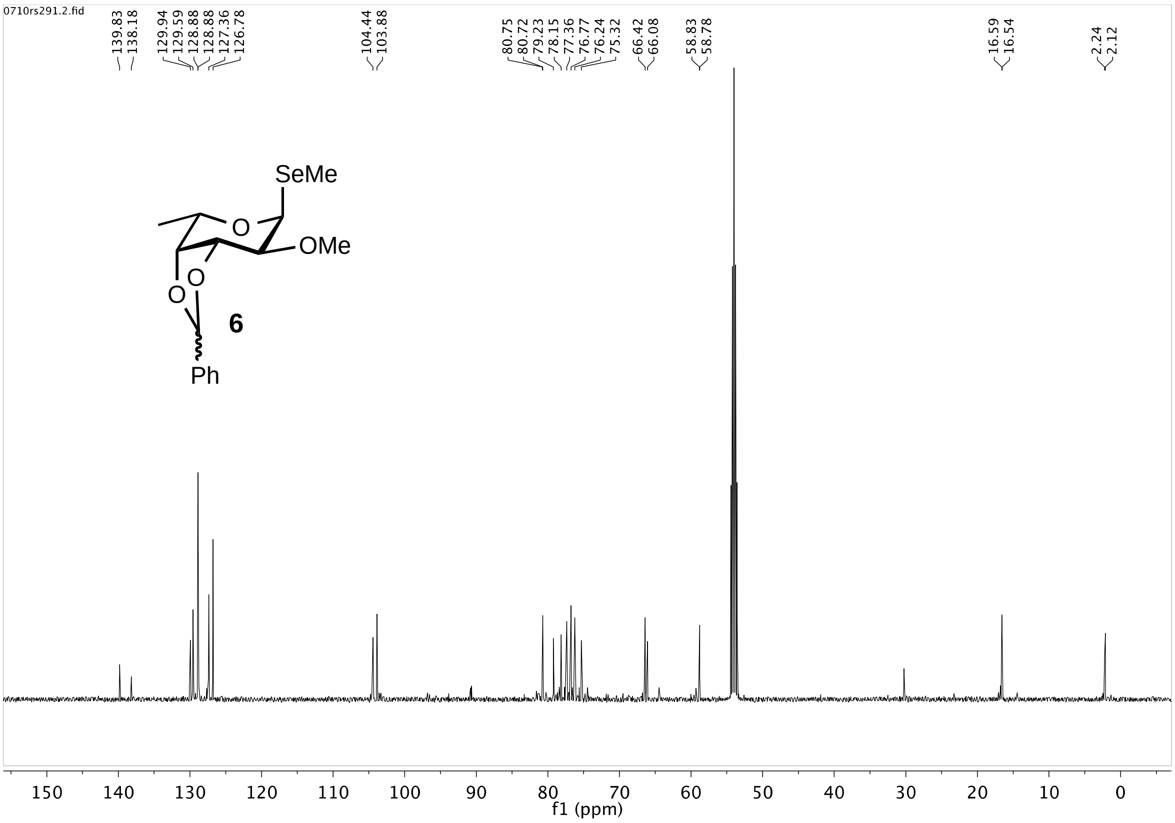

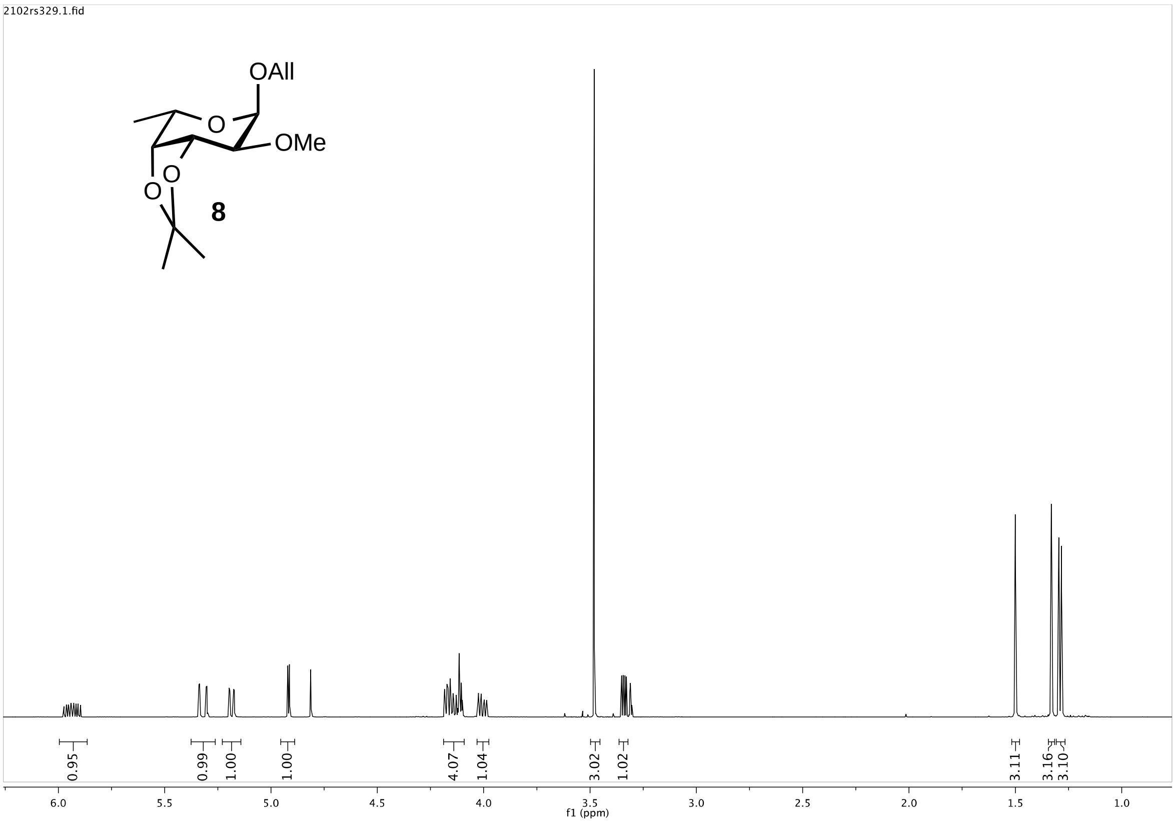
2102rs329.1.fid
OAll
OMe
O
O
O
8
0.95
0.99
1.00
1.00
4.07
1.04
3.02
1.02
3.11
3.16
3.10
6.0 5.5 5.0 4.5 4.0 3.5 3.0 2.5 2.0 1.5 1.0
f1 (ppm)

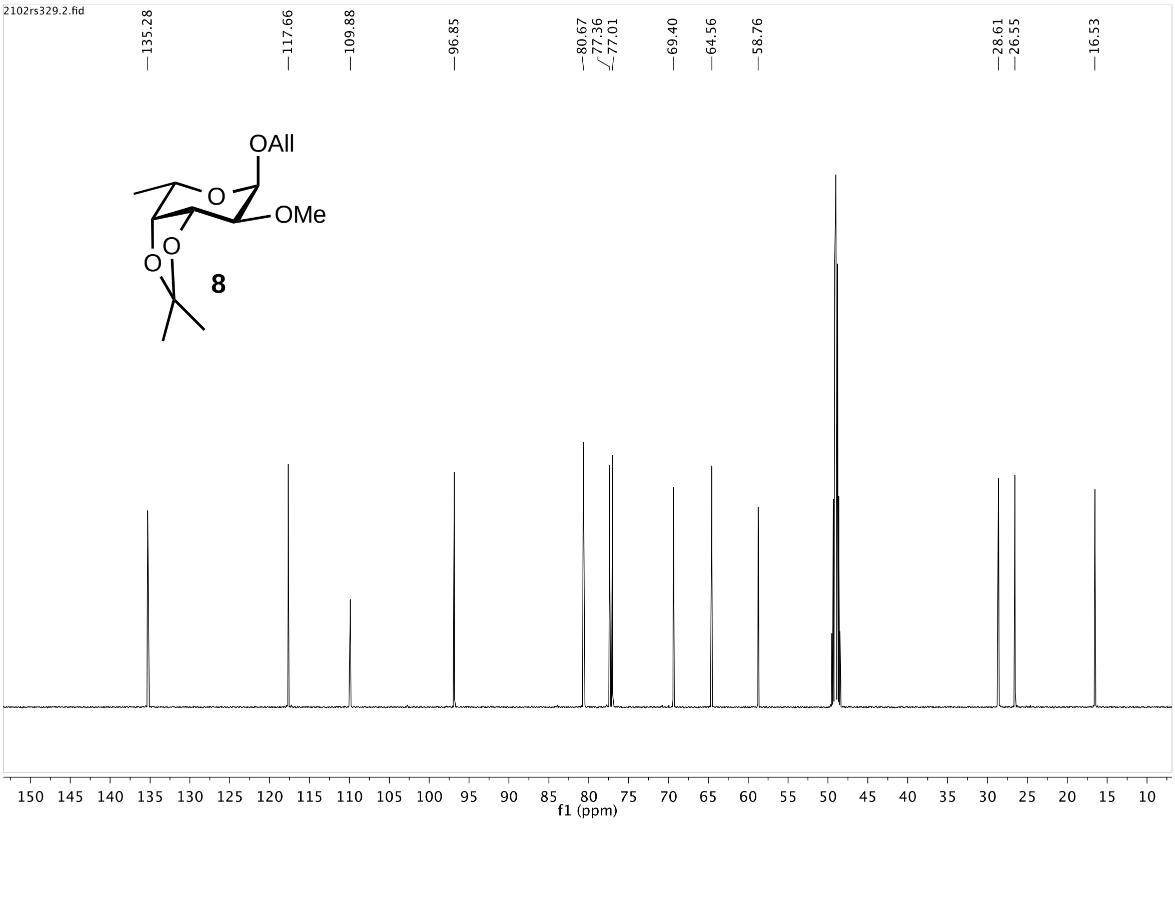
2102rs329.2.fid
135.28
117.66
109.88
96.85
80.67
77.36
77.01
69.40
64.56
58.76
28.61
26.55
16.53
OAll
OMe
O
O
O
8
150 145 140 135 130 125 120 115 110 105 100 95 90 85 80 75 70 65 60 55 50 45 40 35 30 25 20 15 10
f1 (ppm)

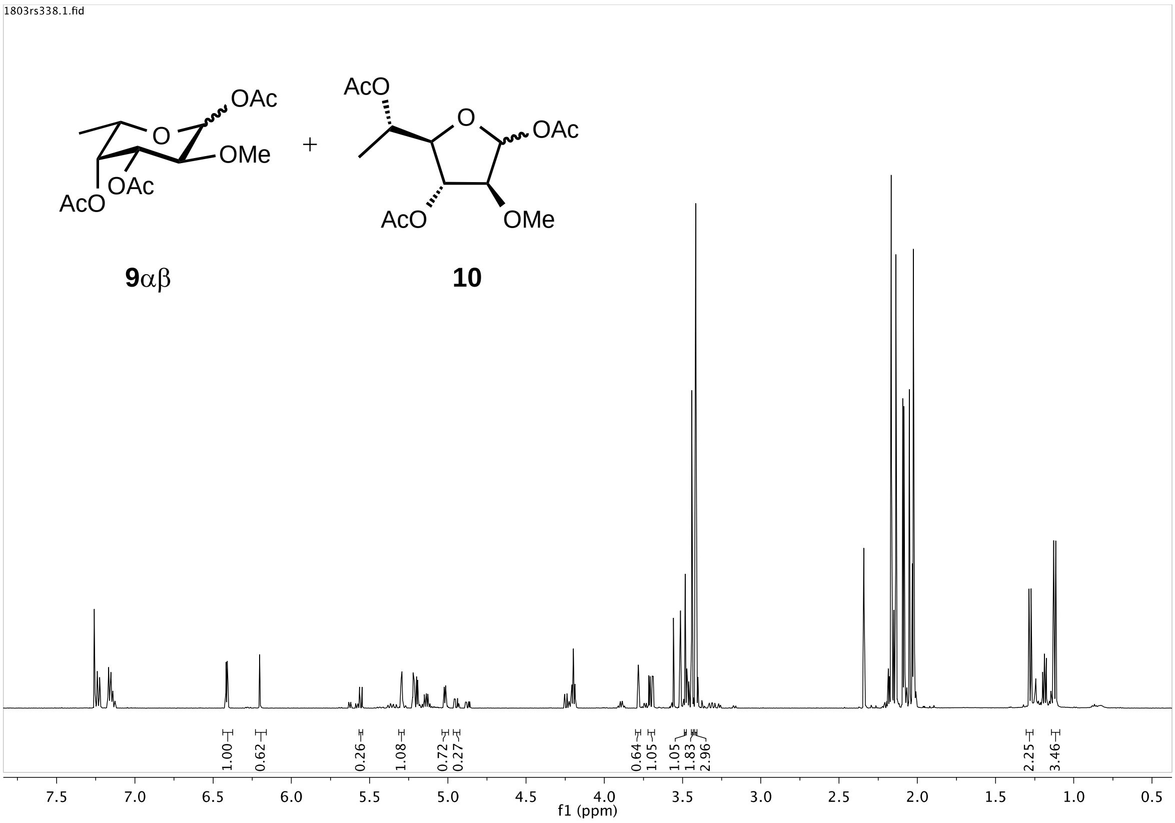
1803rs338.1.fid
OAc
O
OMe
OAc
AcO
+
AcO
O
OAc
AcO
OMe
9αβ
10
f1 (ppm)

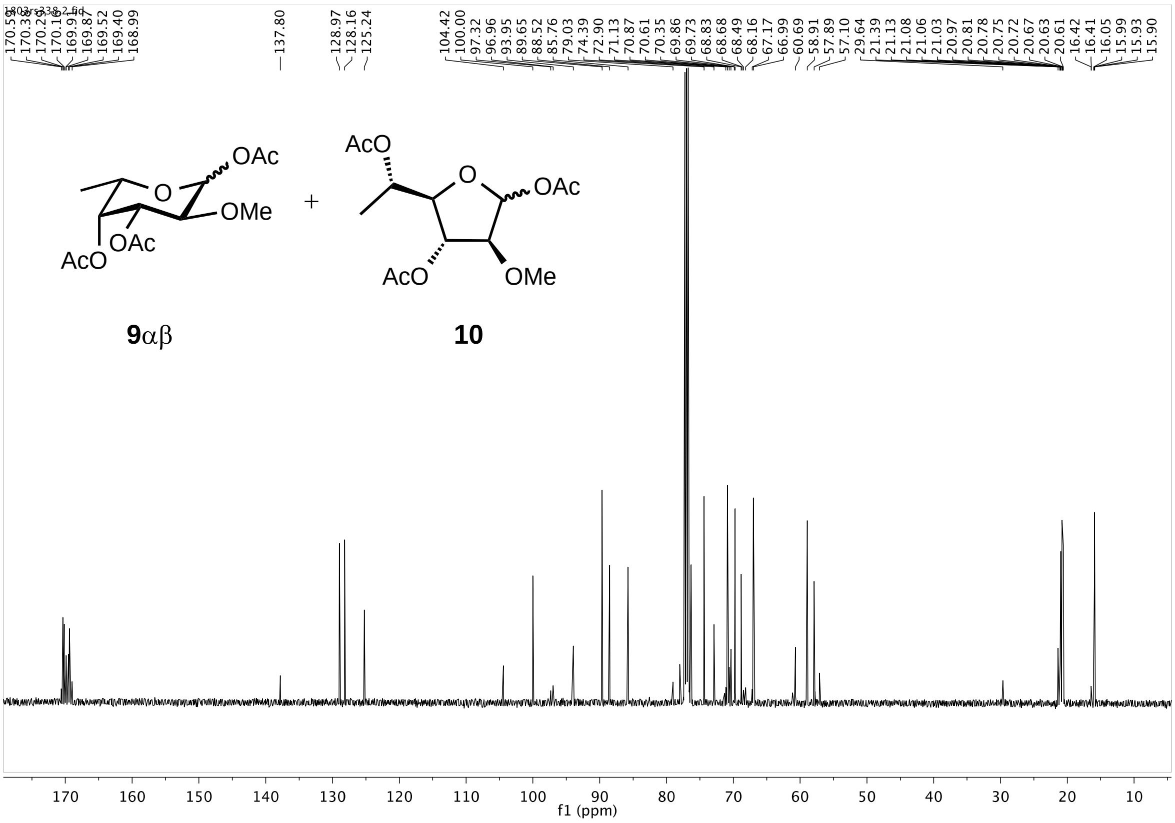
OAc
O
OMe
OAc
AcO
+
AcO
O
OAc
AcO
OMe
9αβ
10
f1 (ppm)

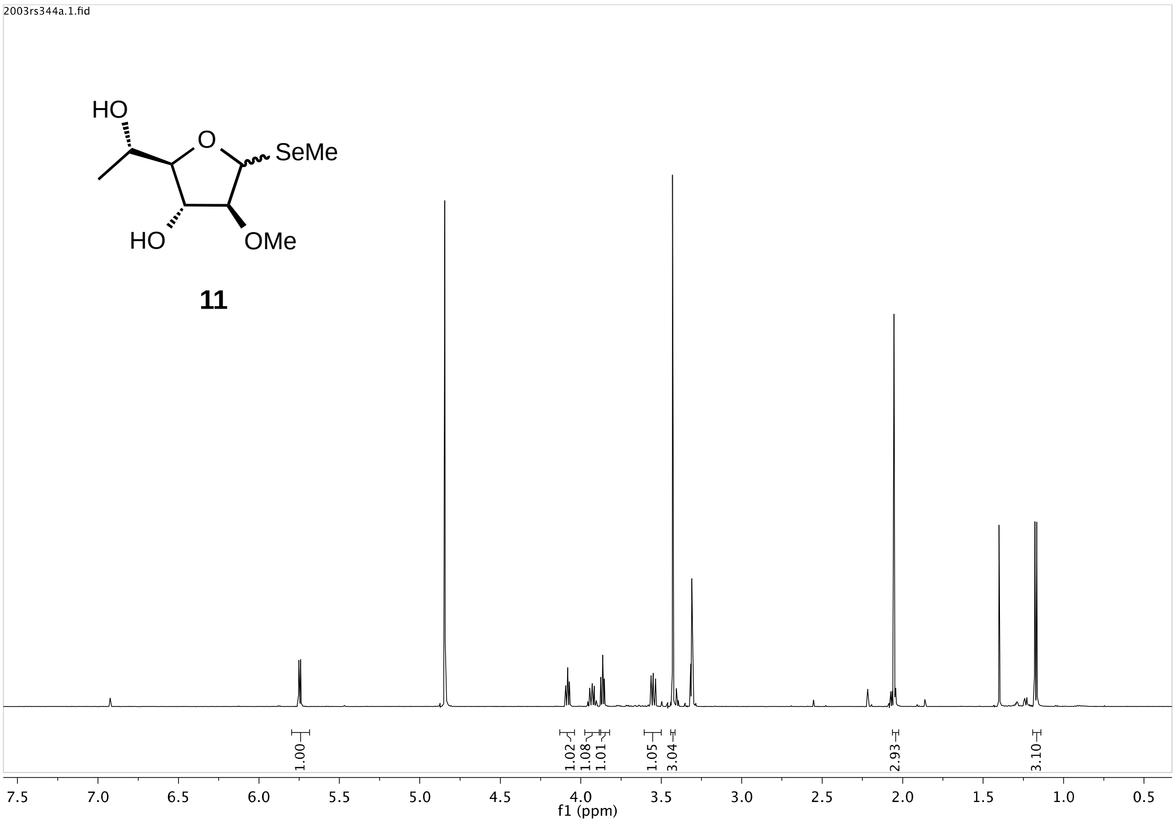
2003rs344a.1.fid
HO
O
SeMe
HO
OMe
11
1.00
1.02
1.08
1.01
1.05
3.04
2.93
3.10
f1 (ppm)

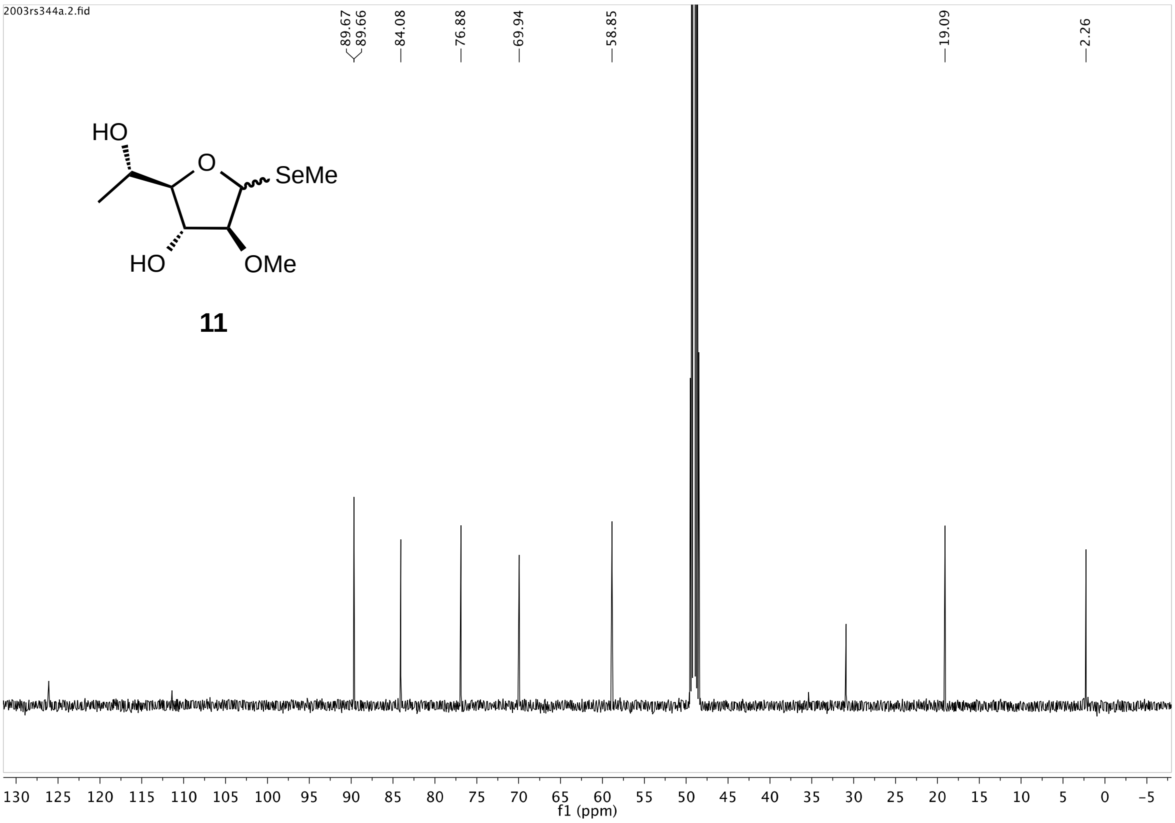
2003rs344a.2.fid
89.67
89.66
84.08
76.88
69.94
58.85
19.09
2.26
HO
O
SeMe
HO
OMe
11
f1 (ppm)

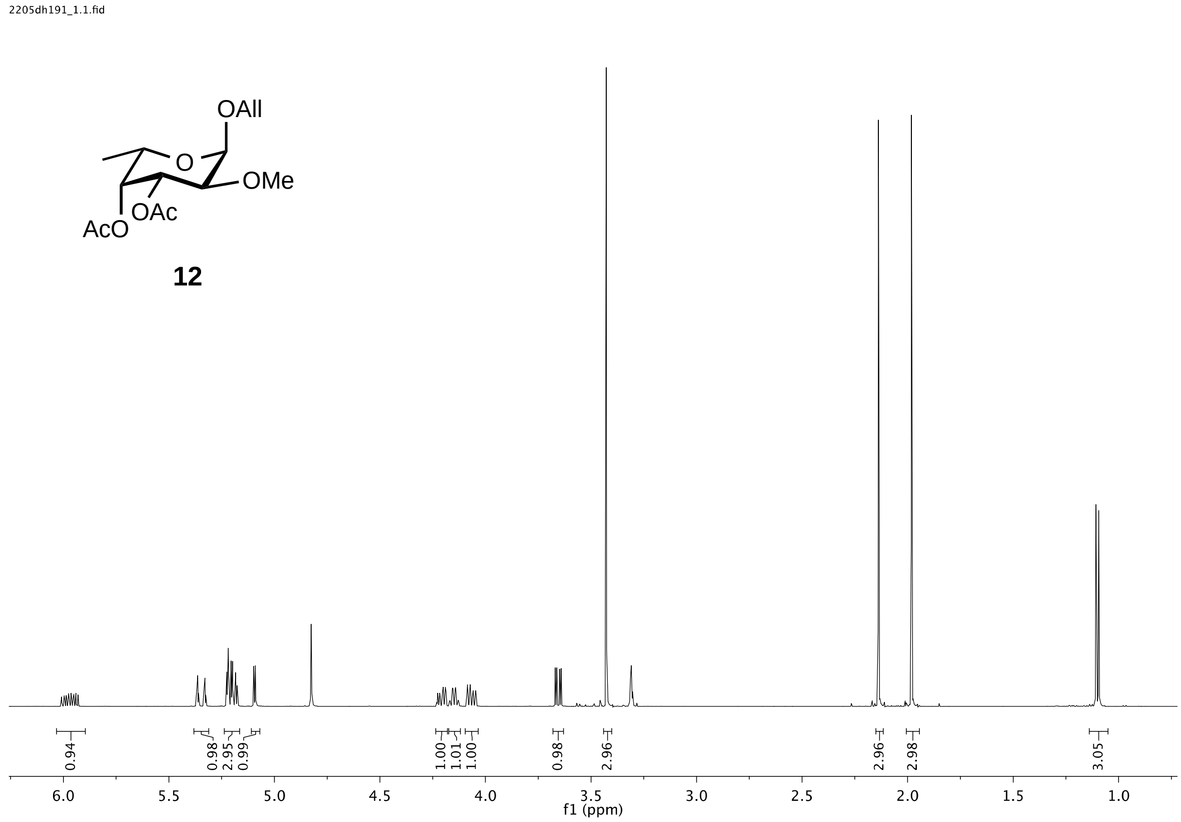
2205dh191_1.1.fid
OAll
O
OMe
OAc
AcO
12
0.94
0.98
2.95
0.99
1.00
1.01
1.00
0.98
2.96
2.96
2.98
3.05
6.0 5.5 5.0 4.5 4.0 3.5 3.0 2.5 2.0 1.5 1.0
f1 (ppm)

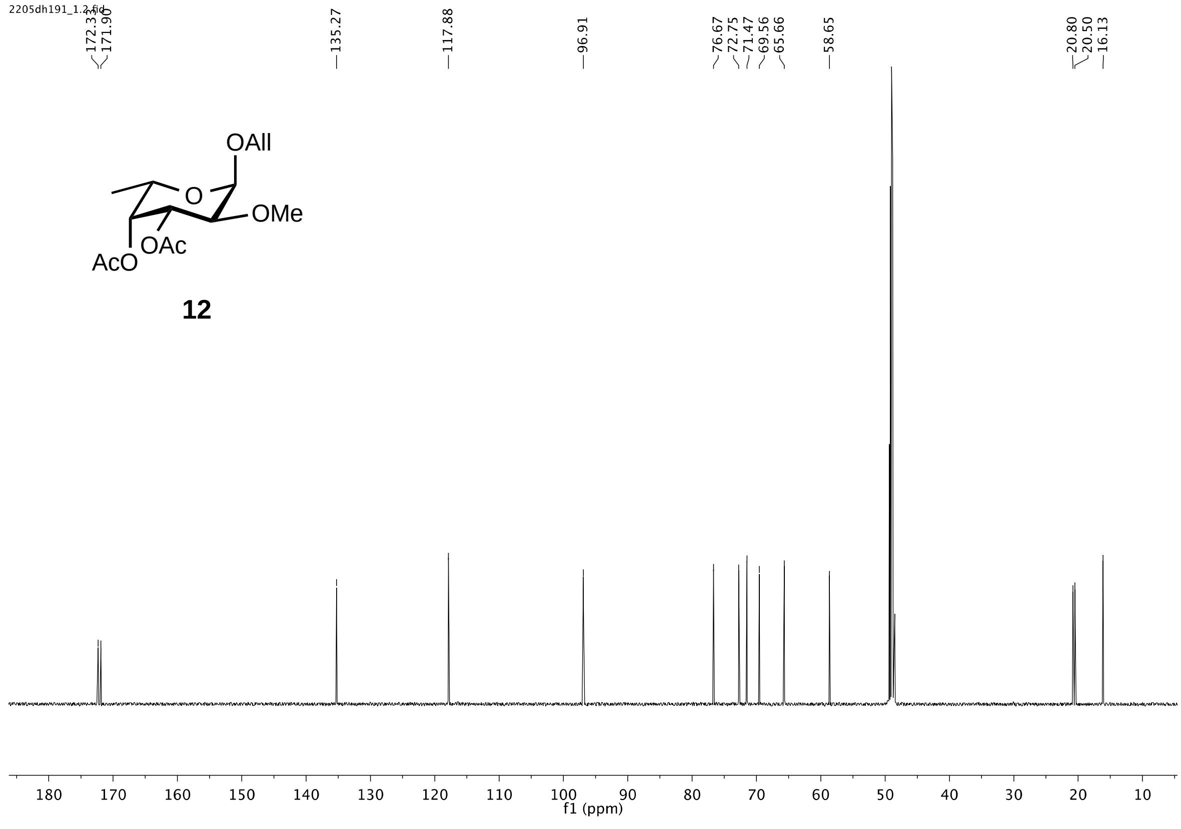
2205dh191_1.2.fid
172.31
171.90
135.27
117.88
96.91
76.67
72.75
71.47
69.56
65.66
58.65
20.80
20.50
16.13
OAll
O
OMe
OAc
AcO
12
180 170 160 150 140 130 120 110 100 90 80 70 60 50 40 30 20 10
f1 (ppm)

2306dh196fr1.1.fid

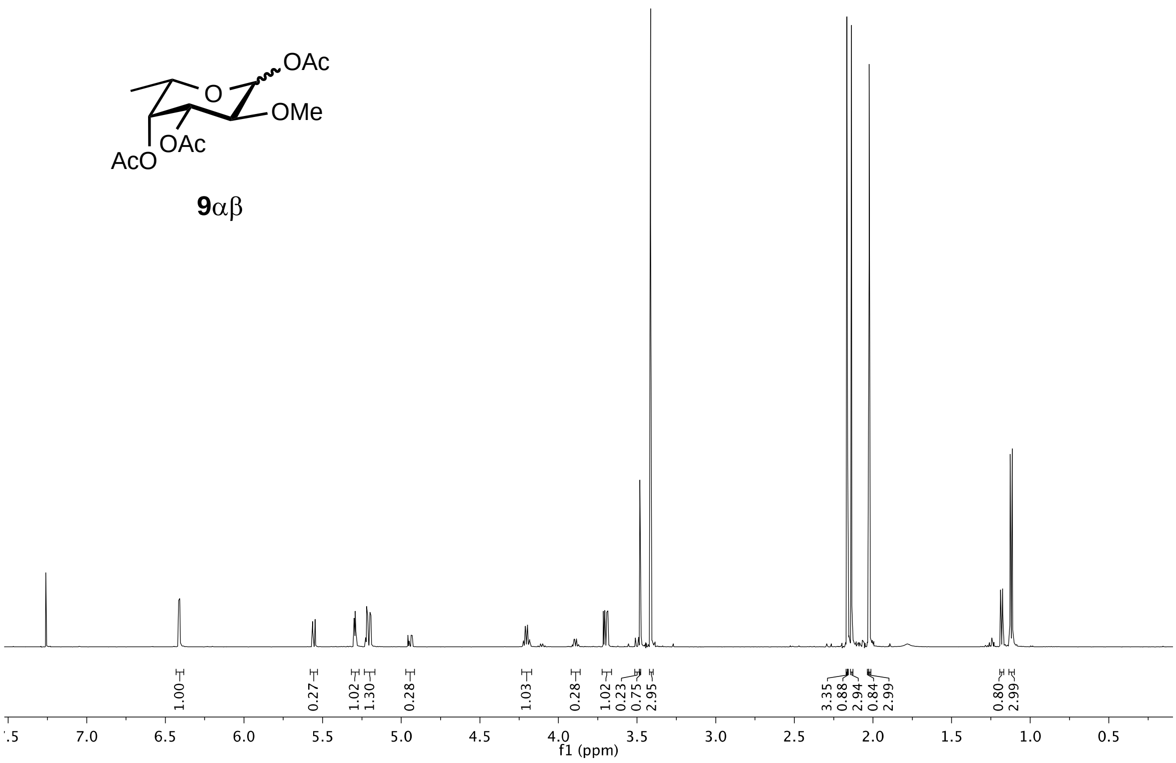

2306dh196fr1.2.fid

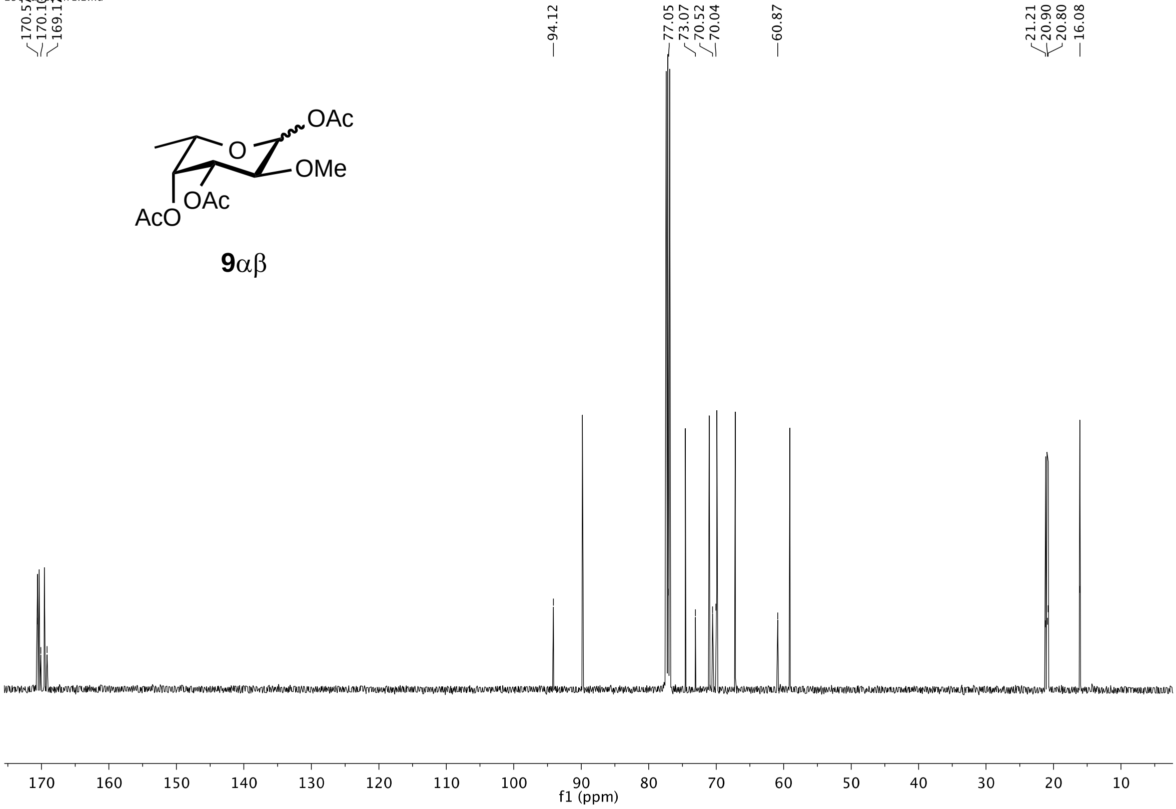

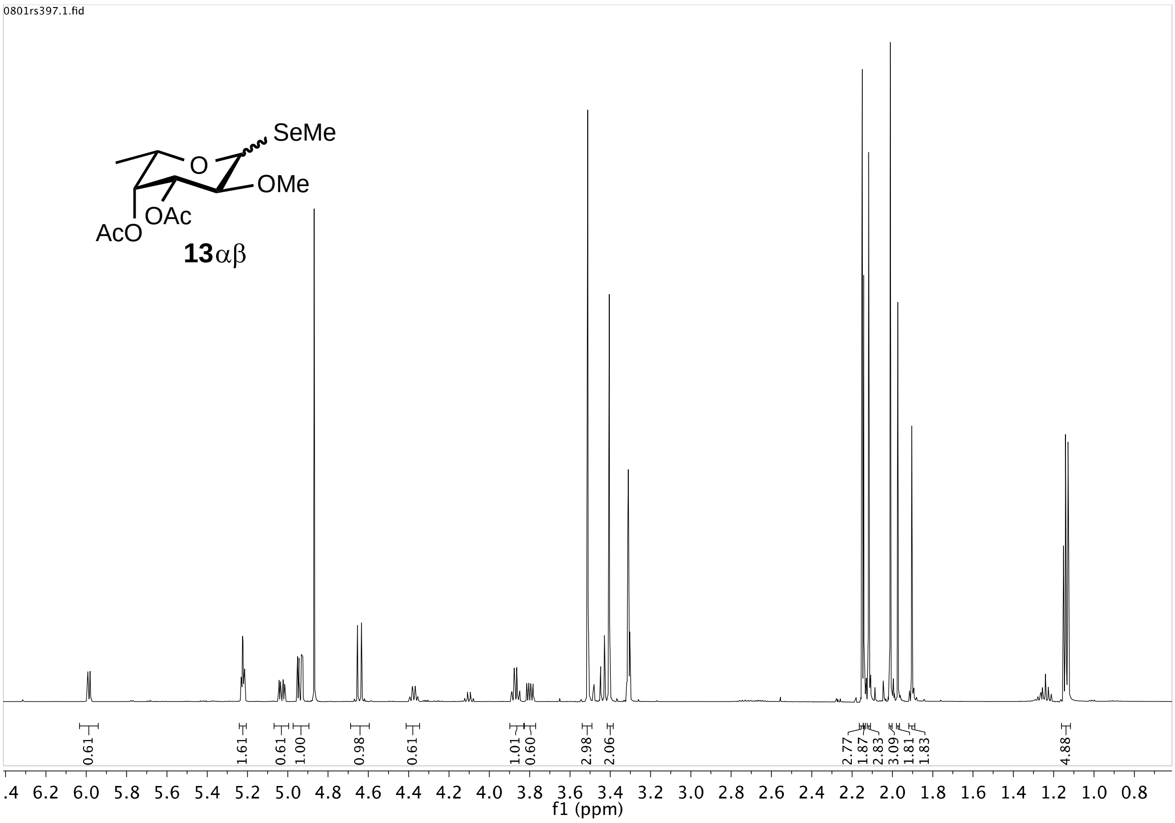
0801rs397.1.fid
SeMe
O
OMe
OAc
AcO
13αβ
0.61
1.61
0.61
1.00
0.98
0.61
1.01
0.60
2.98
2.06
2.77
1.87
2.83
3.09
1.81
1.83
4.88
f1 (ppm)

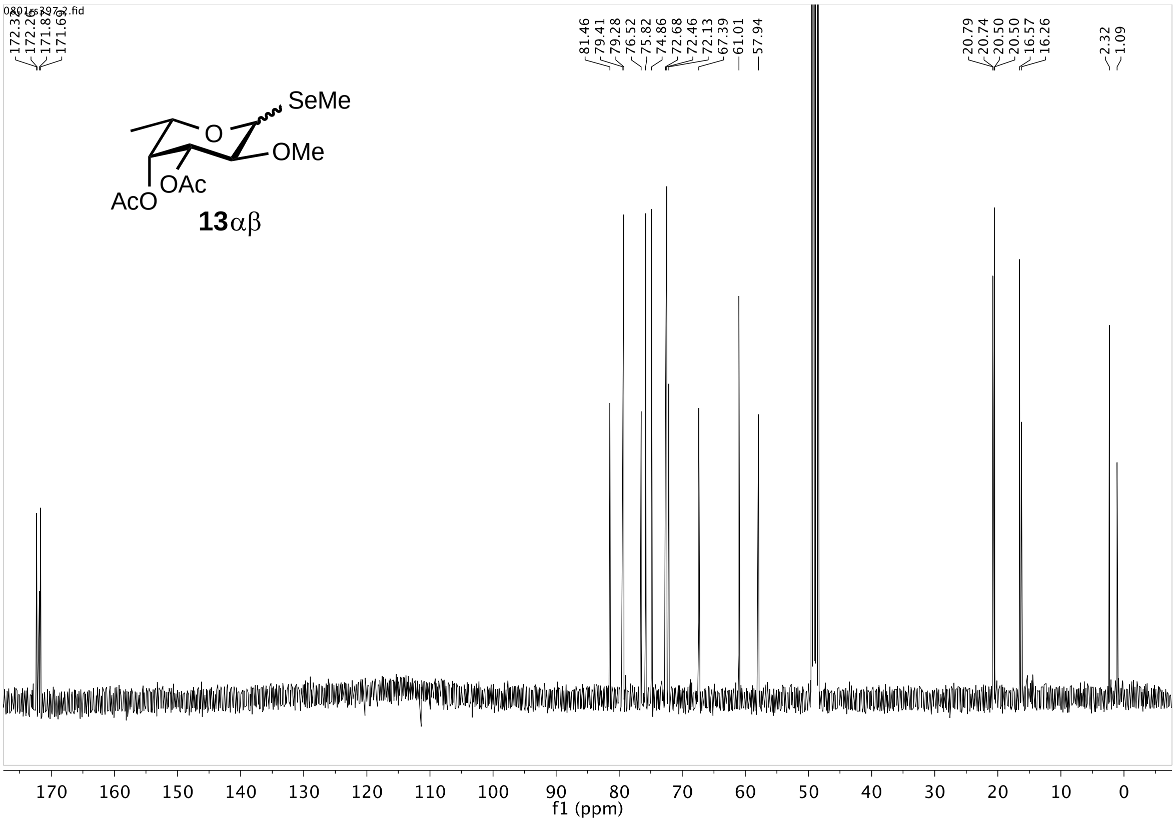
0801rs397.2.fid
172.31
172.26
171.83
171.69
81.46
79.41
79.28
76.52
75.82
74.86
72.68
72.46
72.13
67.39
61.01
57.94
20.79
20.74
20.50
20.50
16.57
16.26
2.32
1.09
SeMe
O
OMe
OAc
AcO
13αβ
f1 (ppm)

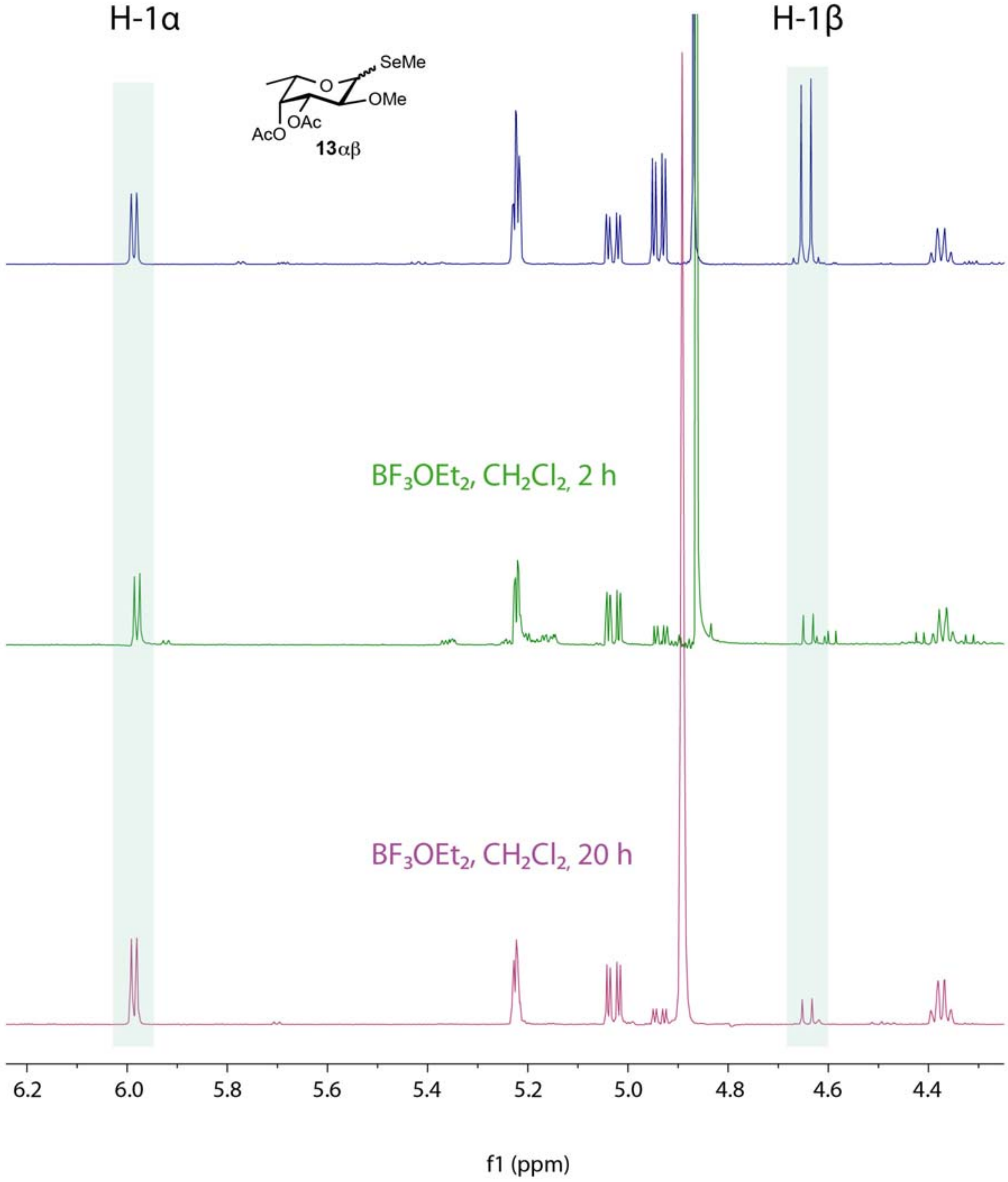
H-1α
H-1β
SeMe
OMe
OAc
AcO
O
13αβ
BF3OEt2, CH2Cl2, 2 h
BF3OEt2, CH2Cl2, 20 h
6.2
6.0
5.8
5.6
5.4
5.2
5.0
4.8
4.6
4.4
f1 (ppm)

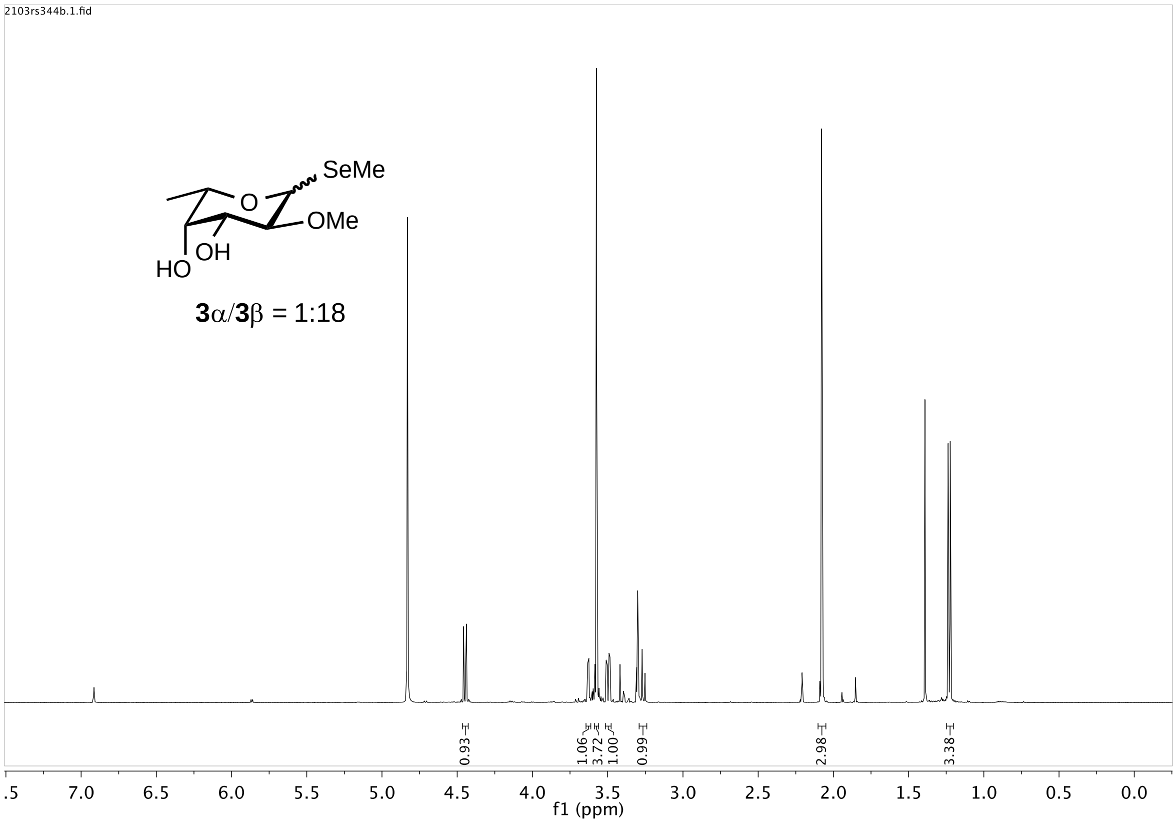
2103rs344b.1.fid
SeMe
O
OMe
OH
HO
3α/3β = 1:18
0.93
1.06
3.72
1.00
0.99
2.98
3.38
f1 (ppm)

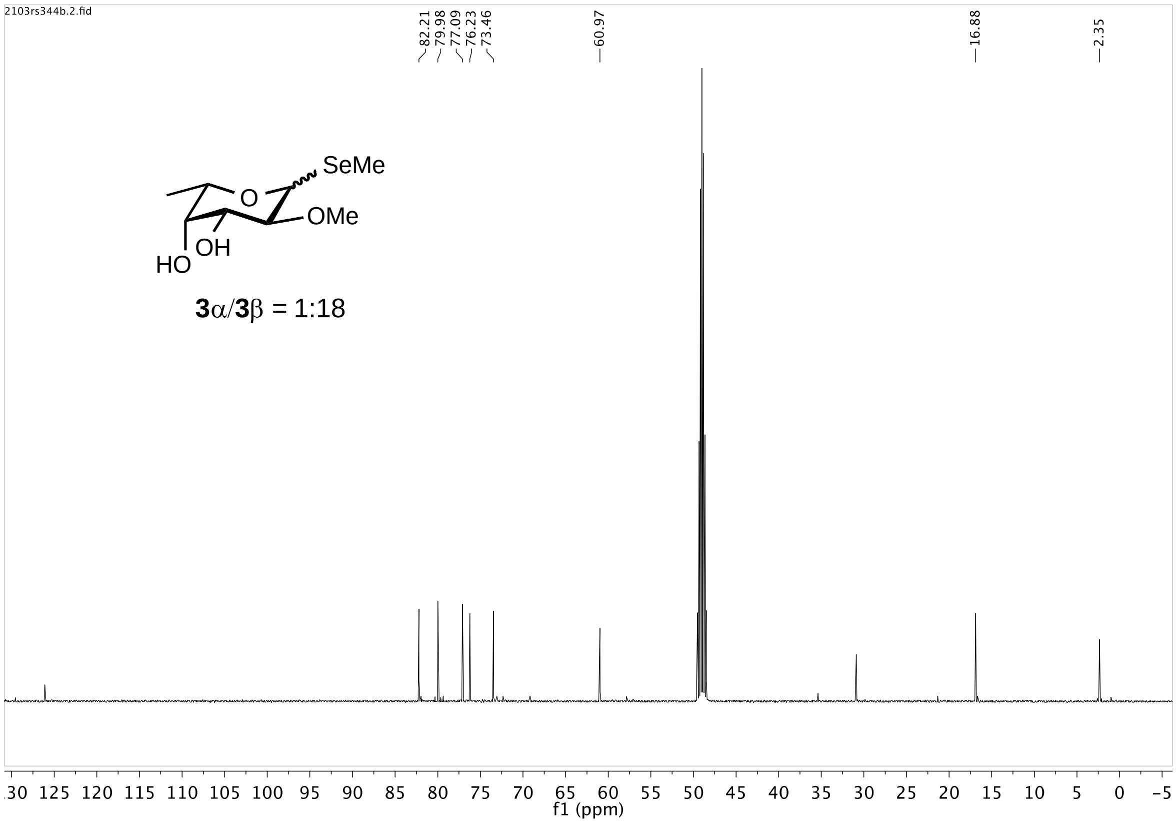
2103rs344b.2.fid
82.21
79.98
77.09
76.23
73.46
60.97
16.88
2.35
SeMe
O
OMe
OH
HO
3α/3β = 1:18
f1 (ppm)

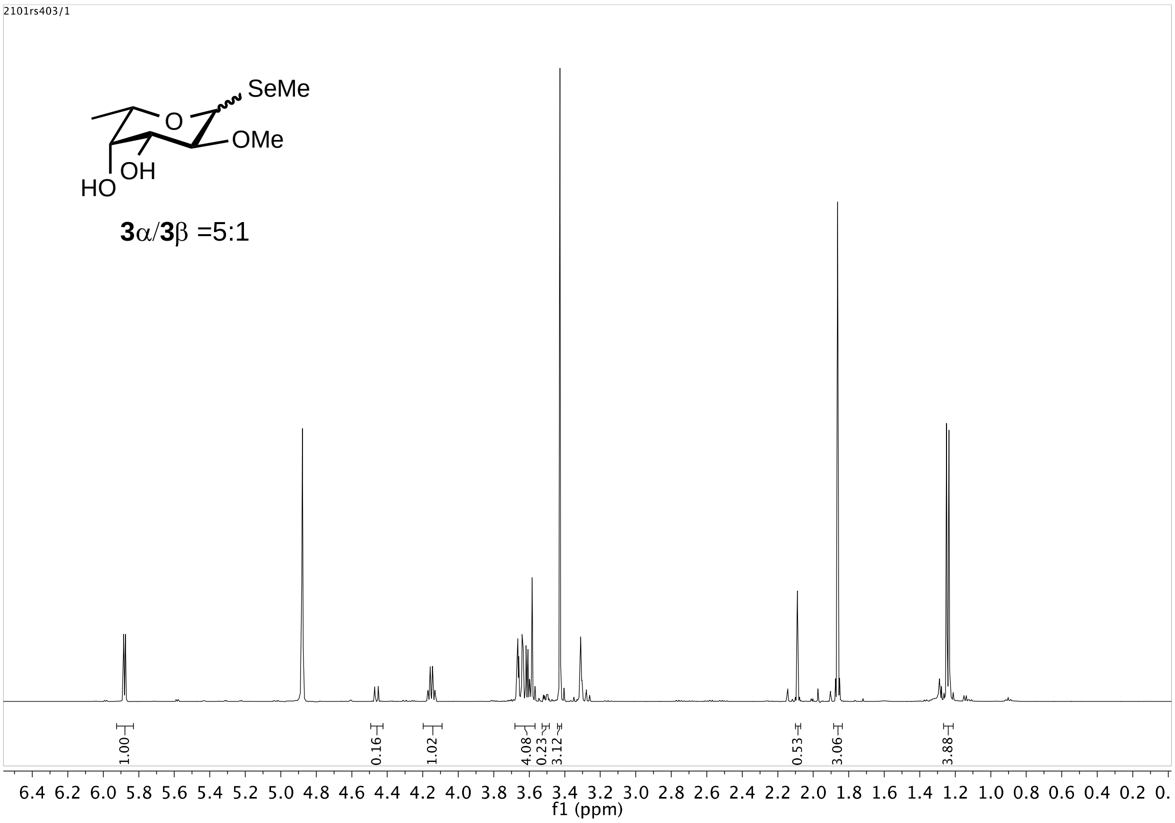
2101rs403/1
SeMe
O
OMe
OH
HO
3α/3β =5:1
1.00
0.16
1.02
4.08
0.23
3.12
0.53
3.06
3.88
6.4 6.2 6.0 5.8 5.6 5.4 5.2 5.0 4.8 4.6 4.4 4.2 4.0 3.8 3.6 3.4 3.2 3.0 2.8 2.6 2.4 2.2 2.0 1.8 1.6 1.4 1.2 1.0 0.8 0.6 0.4 0.2 0.
f1 (ppm)

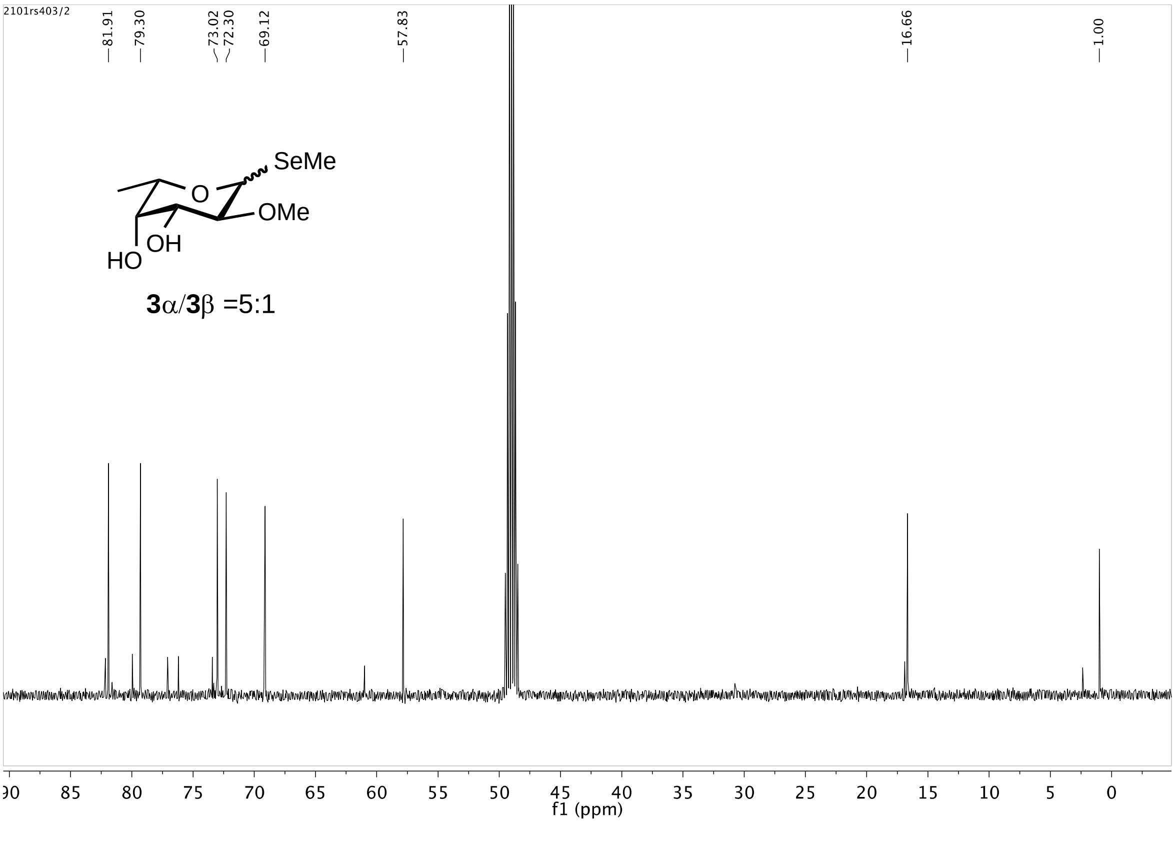
2101rs403/2
81.91
79.30
73.02
72.30
69.12
57.83
16.66
1.00
SeMe
O
OMe
OH
HO
3α/3β =5:1
90 85 80 75 70 65 60 55 50 45 40 35 30 25 20 15 10 5 0
f1 (ppm)
